# A Consistency-Aware Multimodal Sensing Framework for Short-Term Cross-Border Prediction Under Dynamic Trade

**DOI:** 10.3390/s26185806

**Published:** 2026-09-14

**Authors:** Yongyi Wan, Xutong Wang, Xinyue Zeng, Shangshan Chen, Beier Luo, Weijing Yu, Manzhou Li

**Affiliations:** 1 China Agricultural University, Beijing 100083, China; 2 Peking University, Beijing 100871, China

**Keywords:** artificial intelligence-driven sensing, multimodal sensing framework, cross-border prediction, reliability-aware data fusion, cross-modal consistency learning, asynchronous temporal interaction

## Abstract

Short-term cross-border prediction is of significant importance for global supply-chain risk management, international trade decision-making, and stability. However, existing approaches mainly rely on individual price sequences or limited structured variables, making it difficult to effectively perceive complex external factors, including cross-border trade flows, logistics state variations, exchange-rate fluctuations, and policy-driven shocks. To address these challenges, an artificial intelligence-driven sensing-oriented reliability- and consistency-aware multimodal framework for short-term cross-border prediction is proposed. Market conditions, exchange rates, cross-border trade activities, logistics operations, and news–policy events are jointly modeled as multisource intelligent sensing signals. In the proposed framework, the reliability and cross-modal consistency perception module is first developed to dynamically evaluate the credibility of different data sources and suppress the interference caused by missing, delayed, and conflicting information. Subsequently, the asynchronous cross-border temporal interaction module is introduced to capture the time-dependent propagation relationships among trade, logistics, exchange rates, and states. Furthermore, the dynamic trade and policy event perception module is constructed to identify short-term disturbances induced by tariff adjustments, trade restrictions, port disruptions, and major international events, thereby enabling intelligent prediction under complex cross-border environments. Based on a multisource cross-border sensing dataset constructed from January 2022 to December 2025, the performance of the proposed framework is systematically evaluated through three tasks, including short-term direction prediction, volatility forecasting, and risk level prediction. Experimental results demonstrate that the proposed method achieves an accuracy of 0.842, precision of 0.836, recall of 0.829, macro-F1 of 0.832, and AUC of 0.913 in the short-term direction prediction task, significantly outperforming ARIMA, XGBoost, LSTM, TCN, Transformer, PatchTST, and existing multimodal fusion models. Ablation studies further verify the critical contributions of reliability modeling, consistency constraints, asynchronous temporal interaction, and dynamic event perception modules to improving prediction performance.

## 1. Introduction

With the rapid development of digitalized global trade, cross-border e-commerce, international logistics, and commodity prices, exchange rates, and futures prices have become increasingly interconnected across countries and regions [1]. Dynamics are therefore jointly affected by trade flows, logistics conditions, exchange-rate fluctuations, policies, news, and unexpected events rather than by historical prices alone [2]. For example, port congestion or transportation disruptions in major exporting countries can alter supply expectations and subsequently propagate to commodity futures, exchange rates, and related asset prices [3]. Consequently, short-term cross-border prediction should be viewed as a multimodal sensing task that integrates states with trade, logistics, exchange-rate, and external semantic information, providing support for risk monitoring, price forecasting, and supply-chain coordination [4,5]. Traditional prediction methods mainly rely on statistical models such as ARIMA and VAR to capture temporal autocorrelation and linear dependencies among historical variables [6]. Although interpretable, they are limited in modeling nonlinear relationships and external shocks [7]. Machine learning methods such as Random Forest and XGBoost improve nonlinear modeling capability but still depend heavily on engineered features and have difficulty capturing the temporal propagation of trade, logistics, and policy events [8]. More importantly, existing approaches remain dominated by internal variables, while external signals reflecting real supply–demand conditions are insufficiently exploited, leading to degraded responsiveness and prediction performance during abrupt policy changes, logistics disruptions, and severe fluctuations [9].

Recent years have witnessed the widespread application of deep learning models, including long short-term memory (LSTM), gated recurrent units (GRU), temporal convolutional networks (TCNs), Transformer, and PatchTST, to commodity forecasting [10]. Although these models effectively capture temporal dependencies, most studies still formulate prediction as a single-series forecasting problem and insufficiently exploit external signals from real environments [11]. To address this limitation, some studies have incorporated news, social media, and policy texts through sentiment analysis, pretrained language models, and cross-modal attention, while cross-border trade research has introduced customs records, trade orders, automatic identification system (AIS) trajectories, port operations, and policy information for supply-chain monitoring and risk identification [12]. However, these two research directions remain largely isolated, and existing methods rarely model the complete cross-border transmission process from trade and logistics changes to supply–demand adjustments and subsequent responses [13]. Meanwhile, heterogeneous multimodal fusion remains a fundamental challenge [14]. Trade statistics, logistics trajectories, and textual information differ substantially in temporal granularity, statistical characteristics, and semantic meaning [15,16]. Simple feature concatenation cannot effectively capture cross-modal dependencies and may introduce redundant information and spurious correlations [17]. Moreover, heterogeneous sources often contain missing observations, asynchronous updates, delayed responses, and conflicting signals [18]. For example, logistics activities or news reports may indicate supply risks earlier than prices, while trade statistics may lag behind actual conditions [19]. Therefore, reliability-aware multimodal fusion and asynchronous modeling of trade, logistics, exchange rates, policies, and dynamics remain key challenges for improving short-term cross-border prediction [20]. Recent studies have demonstrated the potential of multimodal semantic modeling for forecasting [21]. Representative frameworks integrate cross-lingual semantic alignment, temporal fusion, multimodal attention, or trend-aware feature fusion to enhance risk perception and prediction accuracy across multiple datasets [22]. Nevertheless, they mainly focus on semantic information fusion and still provide limited modeling of cross-border trade propagation mechanisms, heterogeneous information reliability, and asynchronous cross-modal interactions.

To address the aforementioned challenges, a reliability- and consistency-aware multimodal sensing framework for short-term cross-border prediction is proposed. The proposed framework integrates international conditions, exchange rates, cross-border trade flows, logistics trajectories, port operation states, and multilingual news and policy information into unified cross-border sensing signals, thereby establishing an end-to-end deep learning prediction architecture. Specifically, modality-specific encoders are first employed to extract market, exchange-rate, trade, logistics, and event representations. Subsequently, a reliability- and cross-modal consistency-aware mechanism is constructed to dynamically adjust the credibility of different information sources according to missing observations, update intervals, local noise, source stability, and inter-modal correspondence relationships. Based on these representations, asynchronous propagation relationships among trade, logistics, exchange rates, and responses are captured through continuous-time encoding and multi-scale lag-aware cross-modal attention. Furthermore, a dynamic trade and policy event perception module is developed to identify short-term shocks induced by tariff adjustments, trade restrictions, port disruptions, and major international events. Finally, a context-aware fusion network is employed to dynamically regulate the contributions of heterogeneous signals, enabling joint prediction of direction, short-term volatility, and cross-border risk levels. The main contributions of this study can be summarized as follows.

1. **Reformulation of the prediction paradigm.** The conventional cross-border prediction problem is reformulated as a multimodal sensing and fusion problem jointly driven by conditions, exchange rates, trade activities, logistics operations, and policy events. This reformulation overcomes the limitations of traditional single-modal temporal modeling approaches that mainly rely on historical prices and technical indicators, enabling prediction to incorporate real-world cross-border commodity flows and external environmental changes.
2. **Development of an end-to-end multimodal framework.** A reliability- and consistency-aware multimodal cross-border prediction framework is developed to achieve unified encoding and deep interaction among dynamics, exchange rates, trade states, logistics conditions, and unstructured news and policy information. Through reliability estimation and cross-modal consistency modeling, the negative impacts of missing data, statistical delays, and information conflicts on prediction performance are effectively reduced.
3. **Targeted solutions for cross-border business challenges.** A series of specialized modules, including cross-modal signal reliability and consistency perception, asynchronous cross-border temporal interaction, dynamic event impact perception, and context-aware adaptive fusion, are systematically designed to address key challenges including heterogeneous temporal granularity, delayed information propagation, abrupt event shocks, and dynamically changing modality contributions. In particular, multi-scale lag modeling enables the characterization of cross-border transmission mechanisms among policies, logistics, trade activities, and responses with different propagation speeds.
4. **Comprehensive validation of model effectiveness.** The evaluation reported in this manuscript comprises short-term direction prediction, volatility forecasting, risk classification, and controlled component ablations. These experiments directly test the three prediction heads and the contributions of reliability modeling, consistency constraints, asynchronous interaction, event perception, and adaptive fusion. We do not claim separate cross-country, missing-modality, or event-window experiments that are not explicitly reported in the Section 4.

## 2. Related Work

### 2.1. Short-Term Commodity Prediction

The objective of short-term and commodity prediction is to learn the mapping from current conditions to future prices, returns, volatility, or movements by exploiting historical temporal dependencies [23]. The field has evolved from statistical models to machine learning methods and, more recently, deep learning architectures [24]. Early approaches, such as ARIMA and VAR, modeled linear temporal relationships among historical prices, trading volumes, and exchange rates, offering strong interpretability but limited capability for capturing complex nonlinear dynamics. Machine learning models, including Random Forest and XGBoost, further improved forecasting performance by exploiting nonlinear interactions among structured variables such as prices, technical indicators, order books, and exchange rates [25]. Recent advances in deep learning, including LSTM, GRU, TCN, Transformer, Informer, and PatchTST, have substantially enhanced the modeling of long-range temporal dependencies and nonlinear dynamics [26,27]. Nevertheless, most existing methods still formulate prediction as an isolated time-series forecasting task, relying primarily on historical variables while insufficiently exploiting external leading signals such as cross-border trade activities, logistics operations, and policy changes [28]. Consequently, when conditions are reshaped by tariff adjustments, logistics disruptions, or international policy events, these models often exhibit delayed responses and reduced prediction robustness [29].

### 2.2. Cross-Border Trade, Logistics, and Multimodal Risk Sensing

Cross-border trade and logistics sensing aims to characterize real trade activities by integrating heterogeneous information from transactions, commodity flows, transportation processes, and policy environments, thereby supporting trade condition monitoring, anomaly detection, and supply-chain-risk identification [30]. Because cross-border trade involves substantial data heterogeneity and regulatory fragmentation, individual customs declarations or transaction records cannot fully reflect actual commodity flows [31]. Accordingly, existing studies have expanded from structured trade records to multimodal sources such as customs and shipping records, AIS/GPS trajectories, port operations, logistics delays, commodity images, and policy information, which are increasingly modeled using machine learning, deep learning, and graph-based methods [32]. Recent research has further explored multimodal fusion to jointly model complementary sensing sources [33,34]. Trade records describe nominal commodity flows, logistics trajectories and port states capture actual transportation conditions, while news and policies provide external contextual information [35]. However, existing studies primarily use these signals for fraud detection, abnormal transaction identification, and supply-chain-risk assessment [36], with limited attention to how trade and logistics changes propagate through supply–demand expectations, exchange rates, and cross channels to affect short-term commodity [37]. Therefore, a key research direction is to extend trade, logistics, and policy information from conventional risk detection signals to cross-border sensing signals and integrate them into short-term prediction frameworks.

### 2.3. Multimodal Fusion and Event-Driven Forecasting

Multimodal fusion improves prediction robustness by exploiting complementary information from heterogeneous sources and constructing unified representations through feature fusion, cross-modal attention, and multimodal Transformer architectures. In forecasting, textual information such as news and announcements has increasingly been integrated with time series to enhance event perception, while dynamic and reliability-aware fusion mechanisms further adapt modality contributions according to contextual conditions and data quality. However, general-purpose multimodal fusion remains insufficient for cross-border prediction. First, trade data, AIS-based logistics, exchange rates, news, and prices are naturally sampled at different frequencies, making forced synchronization likely to distort their temporal relationships [38]. Second, heterogeneous sources frequently contain missing, delayed, noisy, or conflicting observations, requiring explicit evaluation of modality reliability and cross-source consistency before fusion [39]. More importantly, different signals affect markets through distinct propagation delays: policy and news events may trigger rapid responses, whereas logistics disruptions and trade-flow changes generally propagate through supply–demand expectations before influencing prices [40]. Existing methods still lack effective mechanisms for jointly modeling such asynchronous cross-modal interactions, information reliability, and event-driven propagation delays [20].

Therefore, beyond conventional multimodal fusion strategies, a reliability- and cross-modal consistency-aware representation mechanism, asynchronous temporal interaction module, and dynamic trade and policy event perception mechanism are introduced. A specialized cross-modal alignment, event impact modeling, and context-aware fusion architecture is constructed, enabling simultaneous integration, exchange-rate, trade, logistics, and news–policy information while evaluating source reliability, identifying cross-modal conflicts, and learning the temporal propagation pathways of cross-border events toward target. Consequently, a unified multimodal sensing framework tailored for short-term cross-border prediction is established.

## 3. Materials and Method

### 3.1. Data Collection

A multimodal heterogeneous sensing dataset tailored for short-term cross-border prediction was constructed by integrating multidimensional information sources, including international states, exchange-rate variations, cross-border trade activities, logistics operations, and news–policy events, as shown in Table 1. The data collection period covered January 2022 to December 2025, encompassing multiple major international trade and commodity categories, and diverse evolution periods. Quotation data were primarily collected from international data platforms, including major stock indices, commodity futures, and related asset transaction records. The collected variables included open price, high price, low price, close price, trading volume, return rate, historical volatility, bid–ask spread, and order-book imbalance indicators. These high-frequency trading characteristics were collected at minute-level intervals to ensure that short-term fluctuations and price movement patterns could be effectively captured. Exchange-rate and cross information were obtained from international foreign exchange databases and publicly available interfaces. The collected information mainly consisted of major currency exchange rates against the US dollar, currency return variations, exchange-rate volatility, overseas indices, and cross spreads, which were utilized to characterize the dynamic interactions across different countries and regions.

Cross-border trade data were collected from international trade statistical databases, public customs databases, and trade information service platforms. Import and export records from 2022 to 2025 were included, containing commodity categories, trade values, trade quantities, origin countries, destination countries, trade growth rates, and import–export imbalance indicators. Since trade statistics generally exhibit relatively low update frequencies, the original publication timestamps were preserved and directly incorporated into model training through continuous-time encoding rather than being artificially replicated into high-frequency intervals. Logistics and port sensing data were primarily collected from the AIS, global vessel trajectory databases, and port operation monitoring platforms. The collected information included vessel coordinates, navigation speed, shipping trajectories, arrival time, departure time, estimated arrival time, port congestion level, container throughput, and transportation delays. AIS data were continuously obtained through vessel broadcasting signals, and port-level logistics states were further extracted through geographic region matching approaches to characterize dynamic variations in real commodity transportation processes. News and policy event data were collected from international news databases, government announcement platforms, and trade policy information websites. The collected events covered trade restrictions, tariff adjustments, economic sanctions, supply-chain disruptions, international conflicts, extreme weather events, and central bank policy announcements. Textual information was processed through multilingual semantic models for event identification, and high-level semantic attributes, including event type, associated countries, related commodities, event intensity, sentiment tendency, and uncertainty level, were further extracted.

For reproducibility, every raw record is associated with a source identifier, instrument or entity identifier, native timestamp, time zone, access route, and preprocessing version. Market and foreign-exchange observations are deduplicated by instrument and exchange timestamp, corrected for daylight-saving transitions, and converted to UTC. Trade quantities and values retain their reported units and are converted only after commodity-code harmonization; AIS/GPS points with invalid coordinates or implausible speeds (e.g., coordinates exceeding geographic boundaries or vessel speeds >40 knots) are removed; and duplicated news items are grouped by normalized URL, headline similarity, and a 12 h entity–event window. Specifically, the market quotation records cover major energy and agricultural futures (crude oil, natural gas, soybeans, corn, and copper) retrieved via Bloomberg Terminal API and Refinitiv Eikon, alongside spot FX rates (EUR/USD, USD/CNY, USD/JPY, and GBP/USD). Bilateral trade records are accessed through UN Comtrade and national customs monitoring interfaces, covering major trading corridors across China, the United States, the European Union, Brazil, and Southeast Asia under six-digit HS codes. Vessel trajectories and port states are collected from MarineTraffic AIS feeds and global port performance trackers, encompassing key trans-shipment hubs including Shanghai, Singapore, Rotterdam, Ningbo–Zhoushan, and Los Angeles. All external APIs and historical bulk archives were retrieved between January 2022 and December 2025 and frozen, with specific endpoint identifiers and field mappings documented in the repository.

The textual event-processing pipeline is implemented based on a multilingual pre-trained language model architecture. Specifically, we employ xlm-roberta-base (accompanied by domain-specific fine-tuning on a curated multilingual financial news corpus, checkpoint revision v1.0) to process incoming news reports and regulatory releases in English, Chinese, and Spanish. Unstructured texts are tokenized using the SentencePiece tokenizer with a maximum sequence length truncated to 256 tokens. The model is fine-tuned using the AdamW optimizer with a learning rate of $2\times 10^{-5}$ across 5 epochs, projecting the final pooling layer into a shared 128-dimensional event embedding space ($D_{e}=128$). Events are categorized into a 7-class taxonomy: tariff adjustments, trade restrictions, economic sanctions, shipping disruptions, port operations, macro monetary policies, and extreme weather shocks. Associated countries and commodities are extracted via named entity recognition (NER) aligned with the ISO 3166-1:2020 country standard [41], and HS commodity categories. Event intensity $I_{t}\in \left[0,1\right]$ is quantified by normalized entity-co-occurrence density and vocabulary salience, while sentiment valence and semantic uncertainty $U_{t}\in \left[0,1\right]$ are derived from the Softmax entropy of the model’s multi-class classification head. Duplicate announcements and minor updates across agencies are resolved using a greedy clustering window of 12 h based on cosine similarity (>0.85) over event embeddings.

### 3.2. Prediction Targets and Causal Temporal Alignment

Let $P_{t}$ denote the tradable mid-price (or the settlement price when a mid-price is unavailable) of the target instrument at prediction time *t*, and let h∈{15,30,60} minutes. The horizon return is $r_{t,h}=\log\left(P_{t+h}/P_{t}\right)$. Direction prediction is a three-class task: downward if $r_{t,h}<-\epsilon$, stable if $|r_{t,h}|\;\leq \epsilon$, and upward if $r_{t,h}>\epsilon$. The value of ϵ must be fixed from the training period and reported with the target-instrument roster. Volatility is the realized root-sum-of-squares of one-minute log returns in (t,t+h], $v_{t,h}=\sqrt{\sum_{j=1}^{h}r_{t+j,1}^{2}}$. Risk prediction uses low, medium, and high labels determined by the 50th and 90th percentiles of $v_{t,h}$ estimated on the training period only; the same frozen thresholds are applied to validation and test data. These definitions ensure that all three labels refer to the same forecast horizon and that no validation/test statistic enters label construction.

Four timestamps are retained for every observation *x*: occurrence time $t_{o}\left(x\right)$, publication time $t_{p}\left(x\right)$, ingestion time $t_{i}\left(x\right)$, and model-availability time $t_{a}\left(x\right)$. We define $t_{a}\left(x\right)=\max\left\{t_{p}\left(x\right),t_{i}\left(x\right),t_{q}\left(x\right)\right\}$, where $t_{q}\left(x\right)$ is completion of any deterministic quality-control step. The formal prediction-time information set is $I_{t}=\left\{x:t_{a}\left(x\right)\leq t\right\}$. Inputs for a sample ending at *t* are constructed exclusively from $I_{t}$; the most recent available observation is joined without backward revision, and its age is supplied to the model. When a statistical release is revised, the revision is stored as a new vintage with its own publication and availability times and is never backfilled into earlier samples. News is available no earlier than its first publication/ingestion time, AIS and port states no earlier than the completed aggregation interval, and customs/trade records no earlier than their public release. All timestamps are converted to UTC before the chronological split. This rule is applied before window construction and augmentation.

### 3.3. Data Augmentation

Cross-border prediction involves heterogeneous data sources, including quotations, exchange rates, trade activities, logistics conditions, and news–policy information, which exhibit substantial differences in sampling frequency, missingness, noise intensity, and event distribution. If a model is trained exclusively on the original samples, excessive dependence may be placed on particular high-frequency or high-quality modalities, resulting in increased sensitivity to missing observations, temporal misalignment, and cross-source conflicts in real-world environments. Therefore, while preserving the principal state and prediction labels of each sample, diversified training instances with different levels of information completeness and cross-modal consistency are constructed through random masking, noise perturbation, temporal shifting, and cross-modal recombination. In this manner, model robustness against missing modalities, asynchronous sampling, local noise, and unexpected events can be enhanced. Let the multimodal input within a temporal window be denoted as $X={X^{m}}_{m=1}^{M}$, where *M* represents the number of modalities and $X^{m}$ denotes the *m*-th cross-border sensing signal. The augmented sample can then be uniformly expressed as

(1) $$\tilde{X}=A\left(X;\theta \right),$$

where A(·) denotes the stochastic data augmentation function, and θ represents augmentation parameters such as the masking ratio, noise intensity, and temporal shift magnitude. To simulate the temporary unavailability of individual information sources frequently encountered in real-world cross-border environments, random modality masking is employed to randomly suppress complete modalities, thereby preventing long-term dependence on a single information source. For the *m*-th modality, the augmented representation is defined as

(2) $${\tilde{X}}^{m}=b_{m}X^{m},\;b_{m}∼\mathrm{Bernoulli}\left(1-p_{m}\right),$$

where $p_{m}$ denotes the masking probability of the corresponding modality, and $b_{m}=0$ indicates that the modality is temporarily removed from the current training sample. Meanwhile, temporal masking is further introduced to randomly mask consecutive temporal segments, thereby simulating interruptions in missing AIS trajectories, or local gaps in exchange-rate observations. Given a temporal mask $M_{t}$, the masked sequence can be expressed as

(3) $${\tilde{X}}_{t}^{m}=M_{t}X_{t}^{m}\;M_{t}\in 0,1.$$

This strategy encourages the model to infer the current state from complementary temporal positions and alternative modalities rather than memorizing isolated local patterns. For continuous numerical signals, including prices, exchange rates, port operation states, and logistics indicators, Gaussian noise is further introduced to simulate sensing errors, statistical revisions, and high-frequency disturbances:

(4) $${\tilde{X}}_{t}^{m}=X_{t}^{m}+\epsilon \;\epsilon ∼N\left(0,\sigma_{m}^{2}\right),$$

where $\sigma_{m}$ is configured according to the fluctuation scale of each modality. Considering the intrinsic asynchronous propagation relationships among trade, logistics, news, and responses, time shifting is simultaneously applied to randomly modify the temporal positions of non-target modalities:

(5) $${\tilde{X}}_{t}^{m}=X_{t+\Delta_{m}}^{m}\;\Delta_{m}∼U\left(-\delta_{m},0\right),$$

Here $\Delta_{m}\leq 0$ is a backward-only offset and $\delta_{m}$ is its maximum magnitude. Restricting the perturbation to earlier, already available inputs prevents temporal augmentation from importing future observations. For data with pronounced domain-specific characteristics, logistics trajectory segment masking and news/event masking are further employed. The former randomly removes consecutive AIS/GPS trajectory segments to simulate vessel-positioning loss, missing signals within port regions, and incomplete transportation trajectories. The latter randomly removes portions of news, policy announcements, or event representations, enabling predictions to remain dependent on trade, and logistics states when event information is incomplete. For a sequence with length *T*, a randomly selected interval $\left[t_{s},t_{e}\right]$ is treated as a missing region:

(6) $${\tilde{X}}_{t}^{m}=0,\;t_{s}\leq t\leq t_{e}.$$

In addition to improving robustness against missing information, cross-modal shuffle is specifically introduced to strengthen cross-modal consistency discrimination. The central idea is to replace one modality with information originating from another training sample at a predefined probability, thereby explicitly constructing inconsistent “trade–logistics”, or “trade–market” pairs. Assuming that $X_{i}^{a}$ and $X_{i}^{b}$ originally belong to the same sample, the perturbed representation is formulated as

(7) $${\tilde{X}}_{i}=X_{i}^{a},X_{\pi (i)}^{b},\;\pi \left(i\right)\neq i,$$

where π(·) denotes a random permutation function. Through this strategy, differences between genuine cross-modal correspondence and artificially induced conflicts can be learned, thereby providing more explicit supervisory information for subsequent reliability and cross-modal consistency modeling. Finally, because tariff adjustments, sanctions, port disruptions, and major international news events account for only a limited proportion of the complete temporal sequence, event-window sampling is adopted to increase the occurrence probability of strong-event windows during training. Let the event time be denoted as $t_{e}$; the interval $\left[t_{e}-\tau_{1},t_{e}+\tau_{2}\right]$ surrounding the event is therefore treated as a prioritized sampling window. This strategy prevents the learning process from being dominated by abundant normal observations and enables the model to sufficiently characterize the dynamic process of “event occurrence–cross-border state variation response”. Through the combined augmentation mechanisms described above, the training data cover complex conditions including modality absence, local noise, asynchronous temporal offsets, cross-source conflicts, and rare events, thereby providing a more robust learning basis for reliability perception, cross-modal consistency estimation, asynchronous temporal interaction, and dynamic event perception.

Augmentation is applied strictly to the training folds after the causal information set $I_{t}$ has been constructed; validation and test samples remain untouched to prevent data contamination. Specifically, random modality masking is configured with modality-specific dropout probabilities of 0.05, 0.10, 0.20, 0.20, and 0.15 for market quotes, foreign exchange, trade flows, logistics indicators, and policy–news events, respectively, reflecting their empirical observation completeness in operational environments. Consecutive temporal masks are applied with segment lengths drawn uniformly from U(5,15) native steps for high-frequency (minute-level) series and U(1,3) steps for lower-frequency records. For continuous numerical features, Gaussian perturbations $\epsilon ∼N(0,\sigma_{m}^{2})$ are injected following robust standardization via training-set median and interquartile range (IQR), where standard deviations are fixed at $\sigma_{m}=0.01$ for market and FX series and $\sigma_{m}=0.02$ for trade and logistics series. To provide explicit supervisory contrast for cross-modal consistency discrimination, cross-modal shuffling is activated with a probability of $p_{\mathrm{shuffle}}=0.10$, drawing replacement candidates strictly from within the same chronological training split. Furthermore, to mitigate extreme class and regime imbalances during major international disruptions, event-centered sampling windows are constructed over $\left[t_{e}-60\;\min,t_{e}+120\;\min\right]$ surrounding identified trade and policy events, with a prioritized sampling factor of 3×.

Random time shifting is strictly causal and input-only. The target modality and all labels remain fixed. For a non-target modality, the shift is sampled as $\Delta_{m}∼U\left(-\delta_{m},0\right)$ rather than from a two-sided interval; $\delta_{m}$ is 5 min for FX, 15 min for events, 30 min for AIS/port/logistics aggregates, and one native publication interval for trade statistics. A shifted value is accepted only if its recorded model-availability time remains no later than *t*; otherwise it is masked. Consequently, neither an observation from (t,t+h] nor a later data vintage can enter the input at *t*, and the label $y_{t,h}$ is never shifted or recomputed.

### 3.4. Proposed Method

#### 3.4.1. Overall

After preprocessing, quotations, exchange-rate and cross information, cross-border trade conditions, logistics operation states, and news–policy events are separately fed into modality-specific encoders to extract exchange rate, trade, logistics, and event representations within a unified latent feature space. Based on these representations, the reliability- and consistency-aware module is first constructed to dynamically estimate the credibility of each modality according to observation completeness, update delay, local fluctuation stability, and source availability. Meanwhile, cross-source consistency relationships among trade–logistics, news–trade, and news–pairs are learned through inter-modal representation interactions. A conflict representation is further generated to suppress abnormal, delayed, or contradictory information, thereby obtaining reliability-weighted multimodal representations. Subsequently, the modality representations are introduced into the asynchronous cross-border temporal interaction module, where continuous temporal information is incorporated to preserve the actual temporal distances among heterogeneous observations. Cross-modal attention mechanisms are employed to learn directional dynamic dependencies among trade activities, logistics conditions, and exchange rates. Furthermore, cross-border propagation characteristics are extracted from instantaneous, short-term, and long-term lag perspectives, and different temporal-scale representations are adaptively integrated through a gating mechanism according to current conditions. Consequently, a temporal representation containing cross-border transmission pathways and propagation delays is obtained. The resulting representation is then jointly combined with news and policy event features and delivered into the dynamic event perception module. The effective impact of external events is estimated according to event intensity, associated countries, related assets, and current trade, logistics, and states. Meanwhile, an event residual correction mechanism is introduced on top of the baseline trend prediction, enabling the model to deviate from normal evolution patterns when tariff adjustments, trade restrictions, port disruptions, or major international events occur. Finally, reliability-aware features, asynchronous propagation representations, event-impact features, and current contexts are jointly input into the context-aware adaptive fusion network. The contribution of heterogeneous information sources is dynamically adjusted according to states, and a unified cross-border representation is generated. This representation is subsequently delivered to three prediction heads for direction, volatility, and risk estimation, enabling joint forecasting of future short-term movements, volatility levels, and risk categories. Therefore, an end-to-end framework integrating multimodal sensing, reliability assessment, asynchronous propagation modeling, event correction, and multi-task prediction is established.

#### 3.4.2. Module I: Reliability- and Consistency-Aware Cross-Border Sensing Representation

To enhance the stability of cross-border multimodal representations under missing observations, delayed updates, and cross-source conflicts, five types of input signals, including market, exchange-rate, trade, logistics, and event information, are denoted as $X_{t}^{m}$, where m∈{mar,fx,tra,log,evt}.

**Figure 1:**
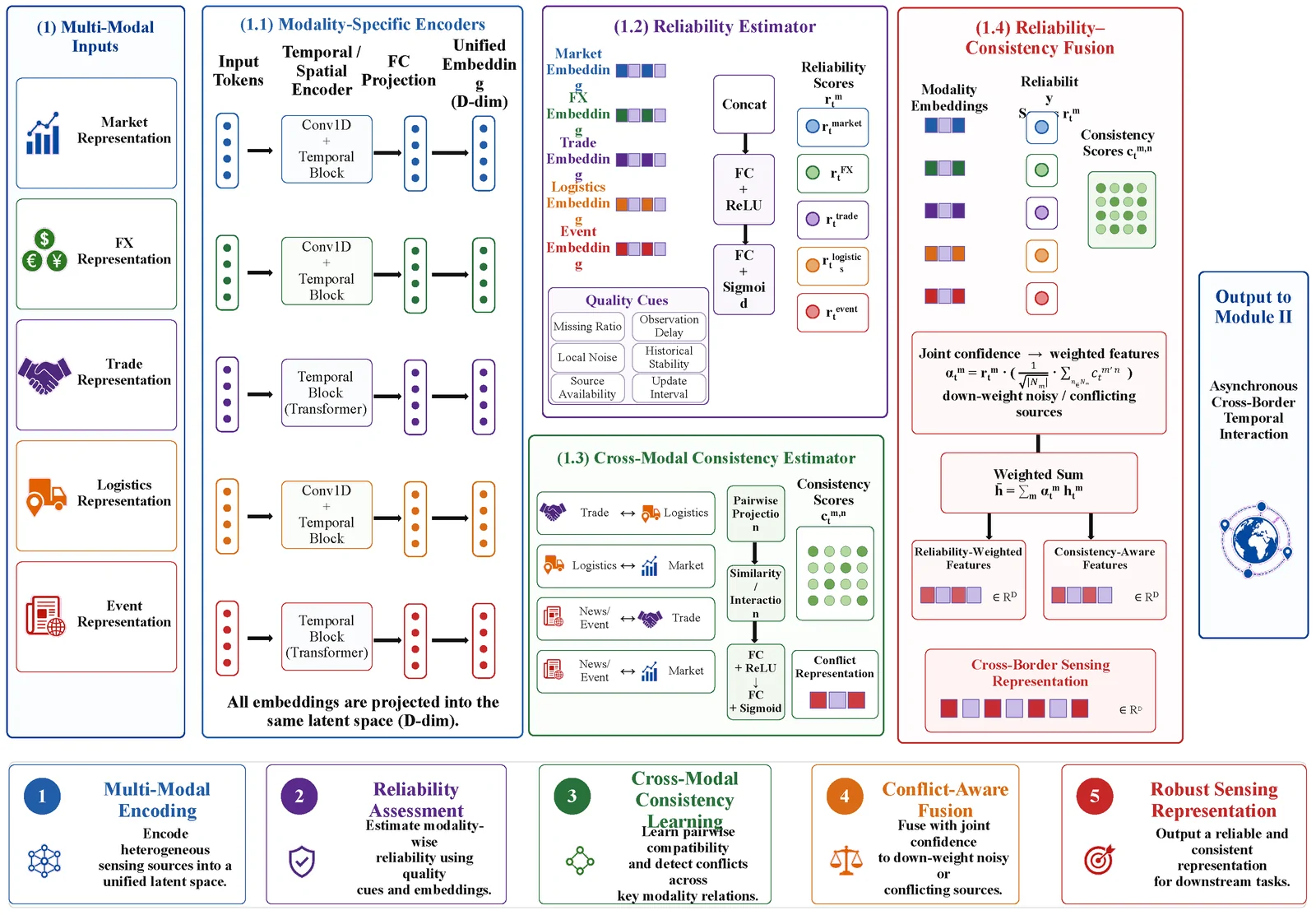
Reliability- and consistency-aware cross-border sensing representation module.

As shown in Figure 1, each modality is processed by an independent three-stage encoder to perform feature mapping. The input feature width, temporal length, and initial channel dimension are denoted as $W_{m}$, $H_{m}$, and $C_{m}$, respectively. The output channels of the three encoding layers are represented as $C_{1}$, $C_{2}$, and $C_{3}$, and all modality representations are further projected into a shared latent dimension *D* through linear transformation. For sequential signals, including exchange-rate and logistics information, one-dimensional convolution and gated residual units are employed to capture local variation patterns. For trade and event representations, two-layer feed-forward projections combined with self-attention mechanisms are utilized for semantic compression. The unified encoding process is formulated as

(8) $$Z_{t}^{m}=\phi_{m}\left(X_{t}^{m}\right)=W_{m}^{\left(3\right)}\sigma \left(W_{m}^{\left(2\right)}\sigma \left(W_{m}^{\left(1\right)}X_{t}^{m}+b_{m}^{\left(1\right)}\right)+b_{m}^{\left(2\right)}\right)+b_{m}^{\left(3\right)},$$

where σ(·) denotes the nonlinear activation function. Subsequently, a reliability estimator is constructed by jointly incorporating modality representations with missing masks $M_{t}^{m}$, observation intervals $\Delta_{t}^{m}$, local noise levels $N_{t}^{m}$, historical stability indicators $S_{t}^{m}$, and source availability measurements $A_{t}^{m}$. A two-layer perception network is employed to estimate the dynamic reliability score:

(9) $$r_{t}^{m}=\mathrm{Sigmoid}\left(W_{r}^{\left(2\right)}\sigma \left(W_{r}^{\left(1\right)}\left[Z_{t}^{m},M_{t}^{m},\Delta_{t}^{m},N_{t}^{m},S_{t}^{m},A_{t}^{m}\right]+b_{r}^{\left(1\right)}\right)+b_{r}^{\left(2\right)}\right).$$

All reliability cues are computed from observations available at or before *t*. Specifically, $M_{t}^{m}$ is the observed/missing indicator vector in the causal input window; $\Delta_{t}^{m}=\min\left\{\left(t-t_{a}\left(x_{\mathrm{last}}^{m}\right)\right)/d_{m}^{95},1\right\}$ is the clipped age of the last available observation normalized by the 95th percentile $d_{m}^{95}$ estimated on the training set; $N_{t}^{m}=\mathrm{clip}\left(\mathrm{MAD}\left(\Delta x^{m}\right)/{\mathrm{IQR}}_{\mathrm{train}}\left(x^{m}\right),0,1\right)$ measures local first-difference noise; $S_{t}^{m}=1-\mathrm{clip}\left(\mathrm{MAD}\left(x^{m}-\mathrm{median}\left(x^{m}\right)\right)/{\mathrm{IQR}}_{\mathrm{train}}\left(x^{m}\right),0,1\right)$ measures historical stability; and $A_{t}^{m}$ is the fraction of expected source updates successfully received during the preceding 30 calendar days. Local noise and stability use the most recent 60 native observations, or all available observations when fewer than 60 exist. Medians, interquartile ranges, clipping constants, and expected update schedules are fitted on the training period and then frozen. No cue uses $x_{t+1}$, a future release, or a retrospectively revised vintage. However, reliability estimation from individual modalities alone cannot determine whether information sources are mutually supportive. Therefore, a cross-modal consistency estimator is further developed. For each correlated modality pair (m,n), both representations are first projected into a shared consistency space. The compatibility relationship is then calculated by jointly considering feature differences, Hadamard interactions, and conditional correlations:

(10) $$c_{t}^{m,n}=\mathrm{Sigmoid}\left(W_{c}\left[\left|P_{m}Z_{t}^{m}-P_{n}Z_{t}^{n}\right|,\left(P_{m}Z_{t}^{m}\right)⊙\left(P_{n}Z_{t}^{n}\right),P_{m}Z_{t}^{m},P_{n}Z_{t}^{n}\right]+b_{c}\right).$$

Accordingly, the overall consistency of each modality is determined by jointly considering other economically related modalities:

(11) $${\bar{c}}_{t}^{m}=\frac{\sum_{n\neq m}a_{mn}c_{t}^{m,n}}{\sum_{n\neq m}a_{mn}+\epsilon },$$

where $a_{mn}$ represents predefined interaction relationships, including trade–logistics, news–trade, and news–associations. To prevent a single modality with relatively high intrinsic quality but significant inconsistency with the overall cross-border state from receiving excessive importance, reliability and consistency scores are jointly mapped into a unified credibility coefficient:

(12) $$q_{t}^{m}=\frac{\exp\left(\eta_{r}r_{t}^{m}+\eta_{c}{\bar{c}}_{t}^{m}-\eta_{f}f_{t}^{m}\right)}{\sum_{j}\exp\left(\eta_{r}r_{t}^{j}+\eta_{c}{\bar{c}}_{t}^{j}-\eta_{f}f_{t}^{j}\right)},$$

where the conflict measurement $f_{t}^{m}$ is determined according to the deviation between a modality and its consistency neighborhood:

(13) $$f_{t}^{m}=\sum_{n\neq m}a_{mn}\left(1-c_{t}^{m,n}\right){∥P_{m}Z_{t}^{m}-P_{n}Z_{t}^{n}∥}_{2}.$$

The final reliability-weighted representation is generated as

(14) $${\tilde{Z}}_{t}^{m}=q_{t}^{m}Z_{t}^{m}+\left(1-q_{t}^{m}\right)G_{m}\left({\bar{Z}}_{t}^{-m}\right),$$

where $G_{m}\left({\bar{Z}}_{t}^{-m}\right)$ denotes the compensation representation constructed from other reliable modalities. Since $q_{t}^{m}$ is normalized through the Softmax function, $q_{t}^{m}\geq 0$ and $\sum_{m}q_{t}^{m}=1$ are guaranteed. Therefore, the fused representation is constrained within the convex hull of multimodal representations, preventing unbounded amplification caused by individual abnormal modalities. Furthermore, assuming that the modality error is represented as $\varepsilon_{m}$ and errors among different modalities are approximately independent, the weighted fusion error satisfies:

(15) $$\mathrm{Var}\left(\sum_{m}q_{t}^{m}\varepsilon_{m}\right)=\sum_{m}{\left(q_{t}^{m}\right)}^{2}\mathrm{Var}\left(\varepsilon_{m}\right).$$

When low-reliability and high-conflict modalities exhibit larger error variances, their corresponding $q_{t}^{m}$ values are automatically reduced through the joint credibility mechanism. Consequently, the upper bound of fusion uncertainty is further reduced compared with fixed-weight fusion strategies. This design enables abnormal information to be adaptively suppressed under conditions such as prolonged AIS signal absence, delayed trade statistics, uncertain news sources, or contradictory cross-border signals, while preserving valuable information when multiple sources provide mutually supportive evidence. Therefore, a stable and economically consistent multimodal representation is provided for subsequent asynchronous cross-border propagation modeling.

#### 3.4.3. Module II: Asynchronous Cross-Border Temporal Interaction

After the reliability-calibrated multimodal representations ${\tilde{Z}}^{m}\in R^{H_{m}\times W_{m}\times C_{m}}$ are obtained from the previous module, the asynchronous cross-border temporal interaction module avoids forcibly mapping heterogeneous sources into identical observation timestamps. Instead, the original temporal characteristics associated with each observation are preserved.

**Figure 2:**
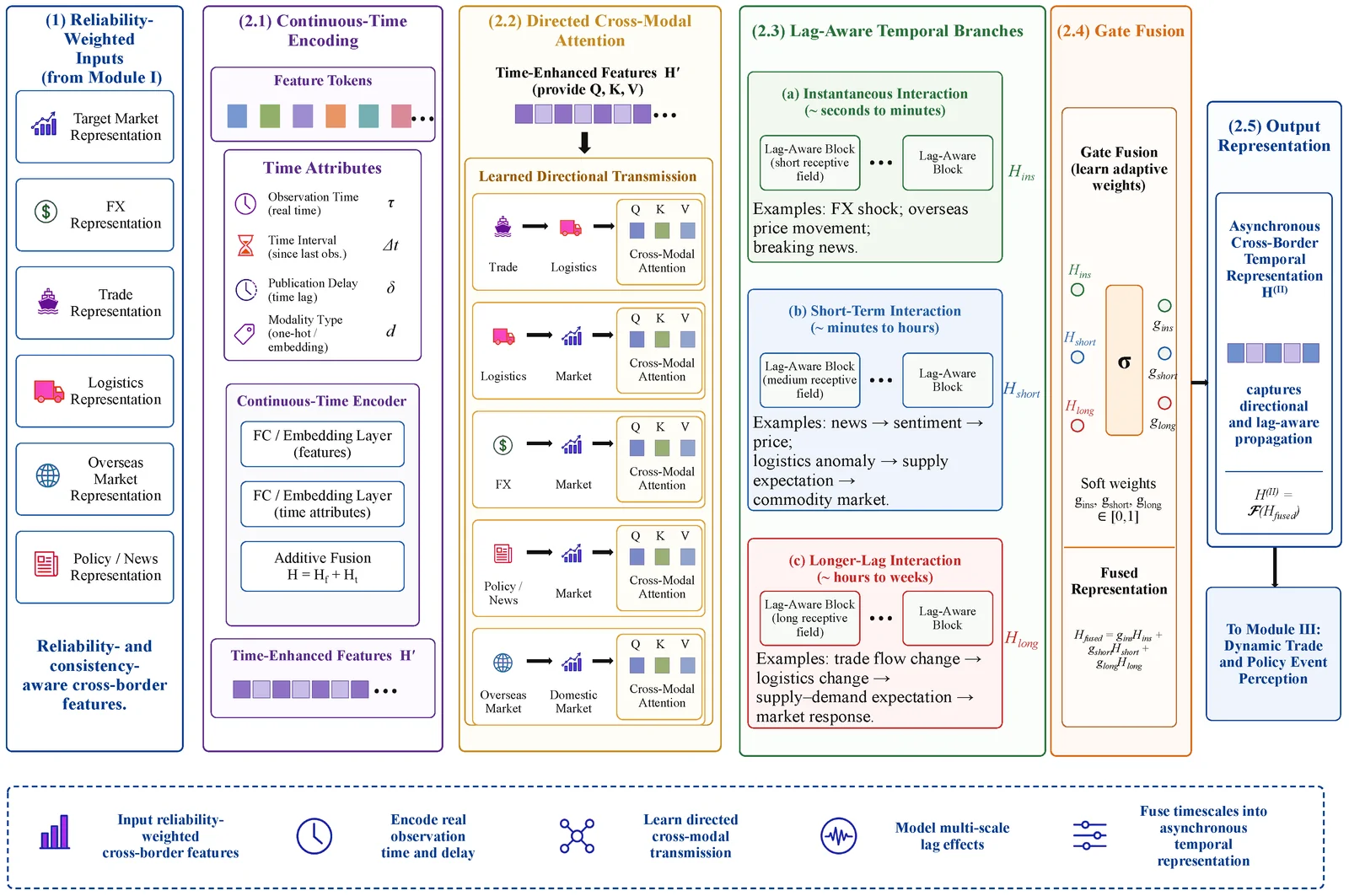
Asynchronous cross-border temporal interaction module.

As shown in Figure 2, a continuous-time projection network with $L_{t}$ layers is first employed to jointly encode feature representations, observation timestamps, adjacent sampling intervals, information publication delays, and modality types. The channel dimension is progressively transformed from $C_{m}$ into the shared temporal representation dimension $C_{t}$. For the *i*-th observation of modality *m*, the continuous-time state is defined as

(16) $$U_{i}^{m}=\mathrm{LN}\left({\tilde{Z}}_{i}^{m}+W_{\tau }^{m}\Psi \left(\tau_{i}^{m},\Delta_{i}^{m},d_{i}^{m}\right)+E^{m}\right),$$

where Ψ(·) represents a learnable continuous-time mapping function, and $\tau_{i}^{m}$, $\Delta_{i}^{m}$, and $d_{i}^{m}$ denote the actual observation timestamp, adjacent observation interval, and publication delay, respectively. Subsequently, a directional cross-modal attention network consisting of $L_{a}$ layers and *K* attention heads is constructed to model propagation relationships among trade-to-logistics, logistics-to-market, exchange-rate-to-market, policy-to-market, and overseas-market-to-target-market interactions. To prevent conventional attention mechanisms from ignoring temporal distances, a learnable temporal decay term is explicitly introduced between query and key representations:

(17) $$e_{ij}^{m\to n}=\frac{{\left(Q_{n}U_{i}^{n}\right)}^{T}\left(K_{m}U_{j}^{m}\right)}{\sqrt{C_{t}}}-\lambda_{mn}\rho \left(\tau_{i}^{n}-\tau_{j}^{m}\right),$$

where ρ(·) denotes a non-negative temporal distance function, and $\lambda_{mn}$ represents the temporal sensitivity coefficient associated with different cross-border propagation pathways. The asynchronous interaction representation is subsequently obtained as

(18) $$A_{i}^{m\to n}=\sum_{j:\tau_{j}^{m}\leq \tau_{i}^{n}}\frac{\exp(e_{ij}^{m\to n})}{\sum_{k:\tau_{k}^{m}\leq \tau_{i}^{n}}\exp\left(e_{ik}^{m\to n}\right)}V_{m}U_{j}^{m},$$

where only observations occurring before the target timestamp are allowed to participate in the attention calculation, thereby preventing future information leakage. Furthermore, three symbolic lag branches are constructed to capture instantaneous, short-term, and long-term propagation patterns, whose receptive fields are denoted as $\Omega_{\mathrm{ins}}$, $\Omega_{\mathrm{sho}}$, and $\Omega_{\mathrm{lon}}$, respectively. Each branch consists of $L_{b}$ lag-aware attention blocks and feed-forward networks with width $C_{b}$: For all three forecast horizons, the lag intervals are fixed in elapsed UTC time as $\Omega_{\mathrm{ins}}=\left[0,15\right]$ minutes, $\Omega_{\mathrm{sho}}=\left(15,60\right]$ minutes, and $\Omega_{\mathrm{lon}}=\left(60,240\right]$ minutes. Each branch uses two lag-aware attention blocks, four heads, and width 128; observations are assigned by their availability time rather than their occurrence time.

(19) $$R_{i}^{\left(s\right)}=\sum_{m,n}\sum_{j\in \Omega_{s}\left(i\right)}\alpha_{ij,s}^{m\to n}V_{s}^{m,n}U_{j}^{m},\;s\in \left\{\mathrm{ins},\mathrm{sho},\mathrm{lon}\right\}.$$

The instantaneous branch captures immediate responses caused by exchange-rate fluctuations, international movements, and sudden news events. The short-term branch models rapid transmission processes, such as news-induced sentiment changes and logistics disruptions affecting supply expectations. The long-term branch focuses on delayed propagation mechanisms, including trade flow variations, logistics evolution, supply–demand expectation adjustments, and subsequent reactions. Finally, a two-layer gating network is utilized to dynamically integrate different temporal scales according to current states:

(20) $$g_{i}^{\left(s\right)}=\frac{\exp\left(w_{s}^{T}\sigma \left(W_{g}\left[R_{i}^{\left(s\right)},U_{i}^{\mathrm{mar}}\right]+b_{g}\right)\right)}{\sum_{r}\exp\left(w_{r}^{T}\sigma \left(W_{g}\left[R_{i}^{\left(r\right)},U_{i}^{\mathrm{mar}}\right]+b_{g}\right)\right)},\;T_{i}=\sum_{s}g_{i}^{\left(s\right)}R_{i}^{\left(s\right)}.$$

Since $g_{i}^{\left(s\right)}>0$ and $\sum_{s}g_{i}^{\left(s\right)}=1$, the fused representation $T_{i}$ remains within the convex combination space of different lag representations, satisfying

(21) $${∥T_{i}∥}_{2}\leq \sum_{s}g_{i}^{\left(s\right)}{∥R_{i}^{\left(s\right)}∥}_{2}\leq \max_{s}{∥R_{i}^{\left(s\right)}∥}_{2}.$$

This inequality demonstrates that the gating mechanism prevents excessive amplification of abnormal responses from individual temporal scales. Meanwhile, because ρ(Δτ) increases with temporal distance, historical observations with larger time intervals naturally receive reduced attention weights under similar semantic relevance. However, signals containing long-term trade transmission information can still be preserved through appropriately learned smaller $\lambda_{mn}$. Therefore, this module is capable of distinguishing immediate impacts from exchange rates and unexpected news, short-term transmission from logistics anomalies, and delayed effects from trade flow variations. By maintaining authentic temporal relationships, an asynchronous propagation representation describing the pathway of trade–logistics–exchange rate–overseas is established, providing temporally directional and cross-dependent information for subsequent dynamic trade and policy event impact modeling.

#### 3.4.4. Module III: Dynamic Trade and Policy Event Perception

After the asynchronous cross-border temporal interaction module, the propagated cross-border state at time *t* is represented as $T_{t}\in R^{H_{t}\times W_{t}\times C_{t}}$. Meanwhile, external events, including tariff adjustments, trade restrictions, sanctions, policy announcements, port interruptions, shipping disruptions, extreme weather conditions, and major international news, are encoded as event tensors $E_{t}\in R^{H_{e}\times W_{e}\times C_{e}}$.

**Figure 3:**
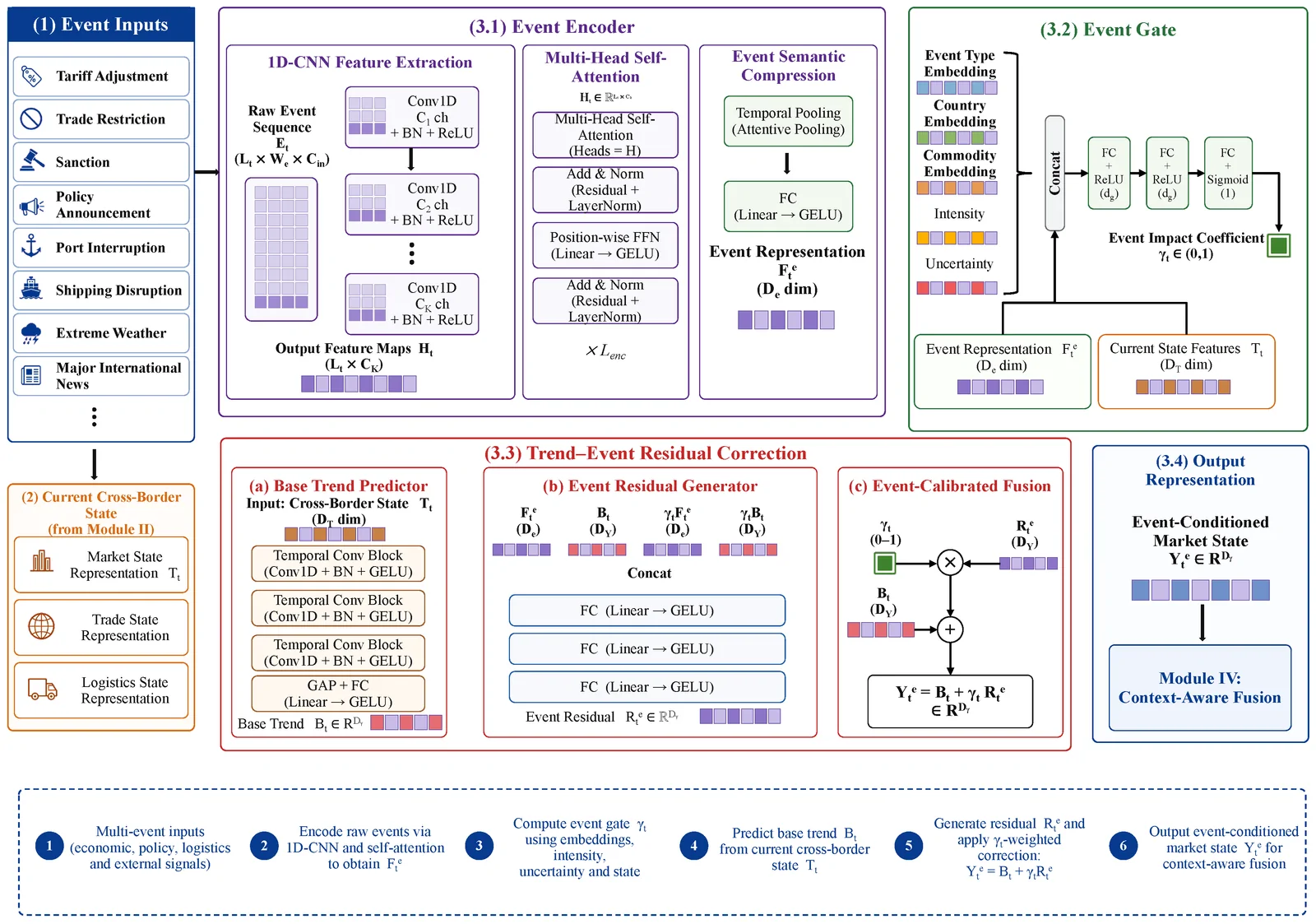
Dynamic trade and policy event perception module.

As shown in Figure 3, the event perception network consists of an $L_{e}$-layer event encoder, an $L_{g}$-layer event gating network, and an $L_{r}$-layer residual correction network. The channel dimension of the *l*-th layer is denoted as $C_{e}^{\left(l\right)}$, and the final event representation is projected into the same latent space dimension $D_{e}$ as the cross-border propagation representation. To avoid estimating event impacts solely according to textual sentiment information, event types, intensity levels, associated countries, related commodities, and uncertainty characteristics are jointly incorporated into the event encoder. The integrated event representation is formulated as

(22) $$F_{t}^{e}=E_{\theta }\left([E_{t},P_{t}^{\mathrm{type}},P_{t}^{\mathrm{country}},P_{t}^{\mathrm{asset}},I_{t},U_{t}]\right),$$

where $E_{\theta }$ denotes the multi-layer event encoding network, and $I_{t}$ and $U_{t}$ represent event intensity and event uncertainty, respectively. Subsequently, a conditional interaction layer is developed to establish correlations between event semantics and the current trade, and logistics propagation states. In this manner, only events with actual relevance to the current cross-border condition can generate significant responses:

(23) $$S_{t}^{e}=\tanh\left(W_{s}F_{t}^{e}+W_{t}T_{t}+W_{x}\left(F_{t}^{e}⊙T_{t}\right)+b_{s}\right).$$

Based on the event-state interaction representation, an event gating network is constructed. The hidden layer widths of the gating network are denoted as $D_{g}^{\left(1\right)},\ldots ,D_{g}^{\left(L_{g}\right)}$, and the event impact coefficient $\gamma_{t}$ is obtained as

(24) $$\gamma_{t}=\frac{1}{1+\exp\left[-G_{\omega }\left(S_{t}^{e},I_{t},U_{t},T_{t}\right)\right]}.$$

Since the Sigmoid function constrains the output within $0<\gamma_{t}<1$, event information is prevented from completely dominating the normal evolution process. Furthermore, a trend–event residual correction mechanism is introduced to explicitly separate normal dynamics from external event-induced disturbances. The baseline trend is first predicted from the cross-border temporal representation:

(25) $$B_{t}=F_{b}\left(T_{t}\right),$$

where $F_{b}\left(\cdot \right)$ represents the baseline trend prediction network. The event residual is then generated by jointly considering the gated event representation and the baseline trend:

(26) $$R_{t}^{e}=F_{r}\left([\gamma_{t}S_{t}^{e},B_{t},B_{t}⊙S_{t}^{e}]\right),$$

where $F_{r}\left(\cdot \right)$ denotes the residual correction network. The final event-adjusted state is obtained as

(27) $$Y_{t}^{e}=B_{t}+\gamma_{t}R_{t}^{e}.$$

The proposed event correction mechanism provides an explicit stability constraint. Assuming that the residual network $F_{r}$ satisfies the Lipschitz condition with constant $L_{r}$, and the event disturbance is represented as $\delta E_{t}$, the variation in the final event-adjusted state satisfies

(28) $${∥\delta Y_{t}^{e}∥}_{2}\leq {∥\delta B_{t}∥}_{2}+\gamma_{t}L_{r}{∥\delta E_{t}∥}_{2}.$$

Because $\gamma_{t}<1$, the influence of event disturbances is bounded after the gating operation. Therefore, weakly correlated news, uncertain information sources, or temporary event noise cannot directly cause excessive deviations in the predicted state. Conversely, when events such as tariff adjustments, economic sanctions, or port disruptions exhibit strong consistency with trade, logistics, and conditions, $\gamma_{t}$ is automatically increased, allowing event residuals to effectively correct the baseline trend. Through this design, different external events are no longer treated equivalently. The stability of normal evolution can be maintained, while significant event-driven shocks with clear cross-border economic transmission effects are effectively captured. Consequently, an event-conditioned state representation is generated and provided to the subsequent context-aware adaptive fusion module for joint prediction of direction, volatility, and risk levels.

#### 3.4.5. Module IV: Context-Aware Fusion and Multi-Task Objective

The fusion inputs are the reliability-pooled sensing representation $H_{t}^{\mathrm{rel}}=\sum_{m}q_{t}^{m}{\tilde{Z}}_{t}^{m}$, asynchronous representation $T_{t}$, event-adjusted representation $Y_{t}^{e}$, and current market context $U_{t}^{\mathrm{mar}}$. After separate linear projections to 128 dimensions, their context-dependent weights are computed by a two-layer gate,

(29) $$\beta_{t}=\mathrm{Softmax}\left(W_{2}\mathrm{GELU}\left(W_{1}\left[H_{t}^{\mathrm{rel}},T_{t},Y_{t}^{e},U_{t}^{\mathrm{mar}}\right]+b_{1}\right)+b_{2}\right),\;F_{t}=\sum_{k=1}^{4}\beta_{t,k}P_{k}H_{t,k},$$

where the gating hidden width is 128 and dropout is 0.2. Separate heads map $F_{t}$ to three-class direction logits, a non-negative volatility estimate (Softplus output), and three-class risk logits. The task losses are cross-entropy for direction, Huber loss for volatility, and class-weighted cross-entropy for risk. Their joint objective uses learned homoscedastic uncertainty weights,

(30) $$L_{\mathrm{total}}=e^{-s_{d}}L_{\mathrm{dir}}+e^{-s_{v}}L_{\mathrm{vol}}+e^{-s_{r}}L_{\mathrm{risk}}+s_{d}+s_{v}+s_{r}+\lambda_{c}L_{\mathrm{cons}},$$

with $s_{d}=s_{v}=s_{r}=0$ at initialization (equivalent to unit task weights) and $\lambda_{c}=0.1$. Class weights for the risk head are calculated from the training fold only. This formulation makes the previously described “dynamic” task weighting explicit and prevents the scale of the regression loss from dominating the two classification losses.

## 4. Results and Discussion

### 4.1. Experimental Configuration

#### 4.1.1. Hardware and Software Platform

All experiments were conducted on a high-performance GPU computing platform. The hardware configuration consisted of an Intel Xeon Gold 6430 processor, an NVIDIA GeForce RTX 4090 GPU with 24 GB memory, 128 GB DDR5 RAM, and a 2 TB NVMe solid-state drive for storing experimental data and model parameters. The GPU was primarily utilized for parallel training and inference of Transformer, PatchTST, Multimodal Transformer, and the proposed framework, while the CPU was responsible for data preprocessing, temporal window construction, and data loading, exchange-rate, trade, logistics, and news–policy information. All baseline models and the proposed method were executed under identical hardware conditions to minimize the influence of computational resource differences on experimental comparisons. The software environment was configured with the Ubuntu 22.04 LTS 64-bit operating system. All models were implemented based on Python 3.10 and PyTorch 2.2, with CUDA 12.1 and cuDNN 8.9 configured for GPU acceleration. Data cleaning, statistical analysis, and feature processing were performed using NumPy, Pandas, and Scikit-learn. Time-series models and traditional machine learning baselines were implemented using Statsmodels, XGBoost, and Scikit-learn, while semantic representations of textual news and policy events were extracted through the Transformers framework. During model optimization, TensorBoard was employed to record variations in loss functions and evaluation metrics, and random seeds of Python, NumPy, and PyTorch were fixed to ensure experimental reproducibility. The complete dataset was divided chronologically into training, validation, and testing subsets with proportions of 60%, 20%, and 20%, respectively, thereby avoiding future information leakage caused by random partitioning. Within the training subset, five-fold temporal cross-validation was further adopted for hyperparameter selection. In each fold, training samples were strictly earlier than validation samples, and the final hyperparameters were determined according to the average performance across five folds. The input window length was set to 60 time steps, and future states were predicted at 15 min, 30 min, and 60 min horizons. The batch size was set to 64, and the maximum number of training epochs was configured as 100. The AdamW optimizer was adopted with an initial learning rate of $\alpha =1\times 10^{-4}$ and a weight decay coefficient of $1\times 10^{-5}$. The hidden feature dimension was set to d=128, the number of attention heads was configured as 4, the number of Transformer encoder layers was set to 3, the hidden dimension of the feed-forward network was 256, and the dropout rate was set to 0.2. During training, a learning rate decay strategy was employed, and early stopping was introduced to terminate training when the validation loss did not improve for 10 consecutive epochs. Meanwhile, the gradient clipping threshold was set to 1.0 to improve optimization stability. For multi-task learning, the initial weights of direction, volatility, and risk prediction losses were all set to 1.0, and were subsequently adjusted dynamically according to task uncertainty. Temporal cross-validation is employed strictly for model selection and hyperparameter tuning. To eliminate potential look-ahead bias and serial correlation leakage, an embargo and purge interval equal to the maximum input window length (60 steps) plus the longest forecast horizon (60 min) is enforced at every chronological split boundary. Once the optimal hyperparameters are identified, the model is retrained on the concatenated training and validation folds (the first 80% of the chronological sequence) and evaluated on the untouched final 20% out-of-sample test period across the 15, 30, and 60 min prediction horizons.

#### 4.1.2. Baseline Models and Evaluation Metrics

Nine representative models were selected as baseline methods, namely ARIMA [42], XGBoost [43], LSTM [44], TCN [45], Transformer [46], PatchTST [47], Early Fusion [48], Multimodal Transformer [49], and cross-modal attention network [50]. ARIMA performs prediction by exploiting autoregressive, differencing, and moving-average relationships within historical sequences, providing strong interpretability with a simple structure. XGBoost utilizes gradient-boosted decision trees to model complex nonlinear relationships and demonstrates strong capability in handling structured features. LSTM captures long- and short-term temporal dependencies through gating mechanisms and is suitable for sequential data with persistent correlations. TCN extracts multi-scale temporal patterns through causal and dilated convolutions while maintaining high parallelization efficiency. Transformer captures global temporal dependencies through self-attention mechanisms and is effective for modeling long-range interactions. PatchTST divides time-series sequences into multiple patches and applies Transformer encoding, enabling joint modeling of local patterns and long-term trends. Early Fusion directly concatenates heterogeneous modality features at the input stage, providing a simple and efficient multimodal integration strategy. Multimodal Transformer employs multimodal attention mechanisms to jointly model, trade, logistics, and textual information, thereby exploiting complementary relationships among different sources. Cross-modal attention network learns association weights among heterogeneous information sources through cross-modal attention, highlighting informative cross-source interactions while suppressing redundant features.

Baseline selection and tuning follow the same temporal folds and early-stopping rule as the proposed model. ARIMA orders are selected by AIC over p,q∈{0,…,5} and d∈{0,1}; XGBoost is tuned over 300–800 trees, depth 3–8, learning rate 0.01–0.1, and subsample/column-subsample 0.7–1.0. LSTM uses one to three layers and hidden width 64–256; TCN uses three or four residual blocks with kernel size 3 and dilations 1,2,4,8; Transformer uses two to four layers, four or eight heads, and width 64–256; PatchTST uses patch lengths 8, 16, or 24 with 50% overlap. Early Fusion, Multimodal Transformer, and the cross-modal attention network receive the identical five modalities, causal windows, preprocessing, and availability masks used by the proposed framework. Unimodal ARIMA, XGBoost, LSTM, TCN, Transformer, and PatchTST receive only the market sequence by design; this distinction is stated explicitly and no multimodal competitor receives privileged features. All search decisions use validation data only.

The evaluation metrics include accuracy, macro-precision, macro-recall, macro-F1, macro-AUC, MAE, RMSE, MAPE, $R^{2}$, PR-AUC, and FPR. For multi-class classification tasks (*K* classes, where K=3 for direction and risk states), the corresponding metric formulations are defined as:

(31) $$\begin{array}{cc} Accuracy & =\frac{\sum_{k=1}^{K}TP_{k}}{N}=\frac{\sum_{i=1}^{N}I\left(y_{i}={\hat{y}}_{i}\right)}{N},\\ Precision & =\frac{1}{K}\sum_{k=1}^{K}\frac{TP_{k}}{TP_{k}+FP_{k}},\\ Recall & =\frac{1}{K}\sum_{k=1}^{K}\frac{TP_{k}}{TP_{k}+FN_{k}}. \end{array}$$

(32) $$F1_{k}=\frac{2P_{k}R_{k}}{P_{k}+R_{k}},\;\mathrm{Macro-F}1=\frac{1}{K}\sum_{k=1}^{K}F1_{k},$$

(33) $$\begin{array}{cc} AUC & =\frac{1}{K}\sum_{k=1}^{K}\int_{0}^{1}TPR_{k}\left(FPR_{k}\right)\;dFPR_{k},\\ \mathrm{PR-AUC} & =\int_{0}^{1}Precision\left(Recall\right)\;dRecall,\\ FPR & =\frac{FP}{FP+TN}. \end{array}$$

(34) $$MAE=\frac{1}{N}\sum_{i=1}^{N}\left|y_{i}-{\hat{y}}_{i}\right|,\;RMSE=\sqrt{\frac{1}{N}\sum_{i=1}^{N}{\left(y_{i}-{\hat{y}}_{i}\right)}^{2}},$$

(35) $$MAPE=\frac{100\%}{N}\sum_{i=1}^{N}\left|\frac{y_{i}-{\hat{y}}_{i}}{y_{i}}\right|,\;R^{2}=1-\frac{\sum_{i=1}^{N}{\left(y_{i}-{\hat{y}}_{i}\right)}^{2}}{\sum_{i=1}^{N}{\left(y_{i}-\bar{y}\right)}^{2}},$$

where *K* denotes the number of target classes (K=3), *N* represents the total number of evaluation instances, and I(·) denotes the indicator function. For each class k∈{1,…,K}, $TP_{k}$, $FP_{k}$, and $FN_{k}$ denote the number of true positives, false positives, and false negatives in a one-vs-rest formulation, respectively; $P_{k}$ and $R_{k}$ represent the class-specific precision and recall, with the reported precision and recall metrics corresponding to their macro-averaged values across all classes. Multi-class AUC is calculated via the one-vs-rest macro-average of individual ROC curves, with $TPR_{k}$ and $FPR_{k}$ denoting the class-specific true positive and false positive rates. For binary risk-alert indicators, FP and TN represent false positives and true negatives. In continuous forecasting, $y_{i}$ and ${\hat{y}}_{i}$ indicate the ground-truth and predicted values, respectively, and $\bar{y}$ denotes the mean value of the ground-truth samples.

### 4.2. Short-Term Cross-Border Direction Prediction

This experiment was designed to evaluate the effectiveness of different methods for short-term cross-border direction prediction and to investigate the influence of multisource cross-border sensing information, temporal dependency modeling, and cross-modal interaction mechanisms on trend identification. Strict temporal cross-validation was adopted, and five evaluation metrics, namely accuracy, precision, recall, macro-F1, and AUC, were employed to comprehensively assess the capability of each model to distinguish future upward, stable, and downward states.

**Figure 4:**
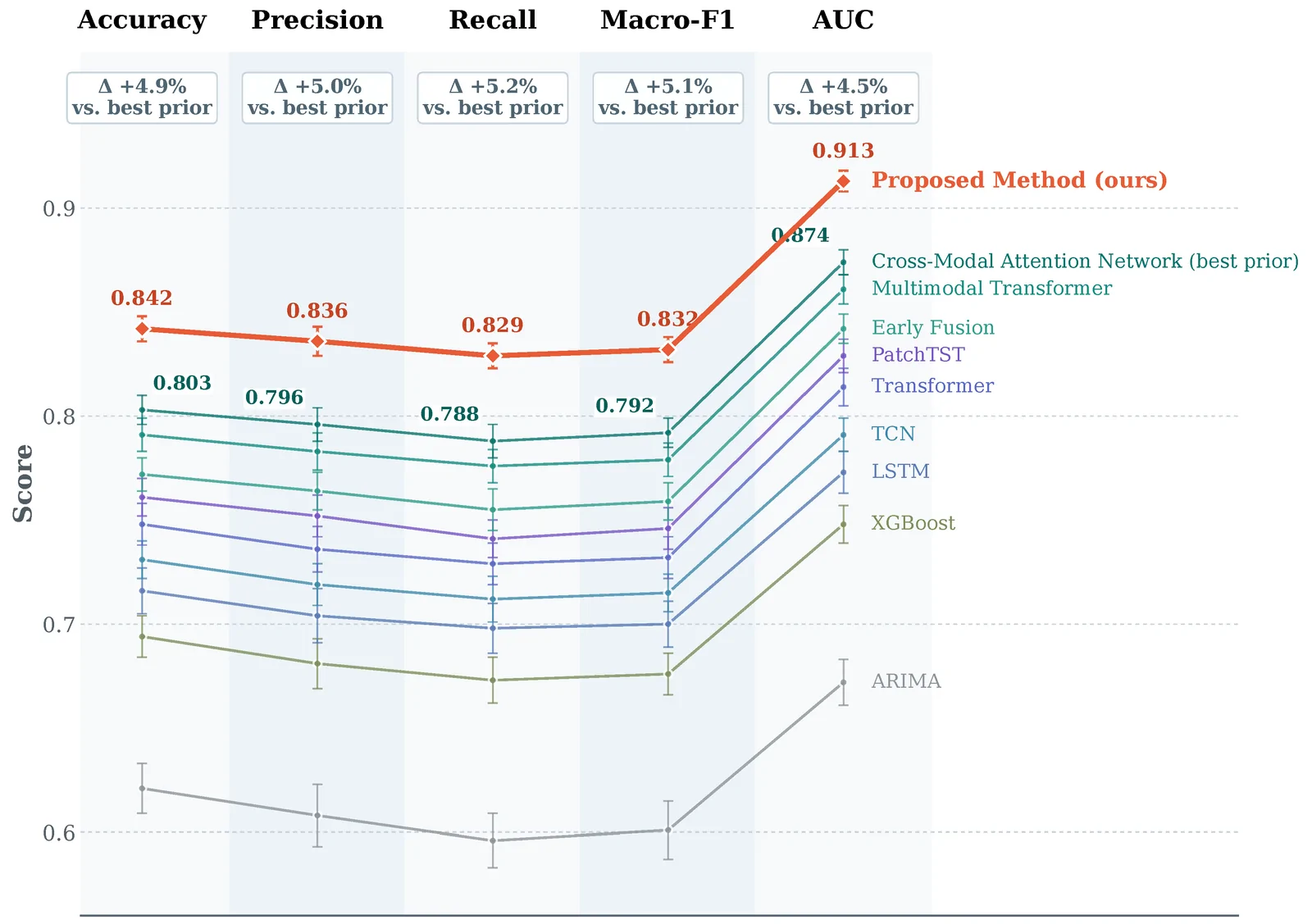
Performance comparison of different methods for short-term cross-border direction prediction.

As shown in Table 2 and Figure 4, the experimental results indicate that the conventional statistical model ARIMA achieved the lowest predictive performance, with accuracy, macro-F1, and AUC values of 0.621, 0.601, and 0.672, respectively. This result can be primarily attributed to the linear autocorrelation assumptions underlying ARIMA. Although periodic variations in relatively stable time series can be captured effectively, nonlinear changes induced by external factors such as cross-border trade flows, logistics states, and policy events remain difficult to model. XGBoost achieved a clear improvement over ARIMA, with accuracy increasing to 0.694, demonstrating that nonlinear combinations based on decision trees are capable of exploiting complex relationships among structured and cross-border variables. Nevertheless, this method largely depends on manually constructed features and lacks explicit capability for modeling continuous temporal propagation processes, which limits its effectiveness in highly dynamic environments. LSTM and TCN further improved prediction performance. LSTM achieved an accuracy of 0.716 and an AUC of 0.773, benefiting from its gated structure for retaining long-term historical information. TCN further increased these metrics to 0.731 and 0.791, respectively, because causal and dilated convolutions expand the temporal receptive field while maintaining computational efficiency, making the architecture more suitable for capturing multi-scale variation patterns in cross-border. Transformer and PatchTST achieved accuracy values of 0.748 and 0.761, respectively, indicating that attention-based architectures can overcome the sequential computation constraints of recurrent structures and identify informative signals across different temporal positions through global dependency modeling. In particular, PatchTST further improves performance by jointly learning local fluctuation patterns and long-term trends through patch-based temporal representations.

Compared with unimodal time-series models, multimodal approaches exhibited stronger predictive capability. Early Fusion achieved an accuracy of 0.772, indicating that the joint use of trade, logistics, and event information provides additional cross-border state information. However, direct feature concatenation cannot effectively distinguish the importance of heterogeneous data sources and remains sensitive to low-quality or conflicting information. Multimodal Transformer further improved accuracy and AUC to 0.791 and 0.861, respectively, demonstrating that multimodal attention mechanisms can effectively exploit complementary relationships among heterogeneous information sources. Cross-modal attention network achieved an accuracy of 0.803 and an AUC of 0.874. Through explicit cross-modal attention weighting, interactions among critical information sources were strengthened, resulting in superior performance compared with conventional multimodal fusion strategies. The proposed method achieved the best predictive performance, with accuracy, macro-F1, and AUC reaching 0.842, 0.832, and 0.913, respectively, corresponding to improvements of 3.9, 4.0, and 3.9 percentage points over cross-modal attention network. These gains are mainly attributed to the reliability, consistency, and asynchronous propagation modeling mechanisms specifically designed for cross-border characteristics. From a structural perspective, conventional fusion methods generally assume comparable contributions among modalities, whereas the proposed framework dynamically reduces the influence of missing, delayed, and noisy information through reliability-aware weighting and identifies genuine relationships among trade, logistics, news, and signals through cross-modal consistency modeling. In addition, the asynchronous temporal interaction mechanism avoids forcibly synchronizing heterogeneous data with different sampling frequencies, thereby enabling the propagation pathway from trade variation to logistics change and subsequent response to be learned more effectively. The dynamic event perception module further enhances responsiveness to tariff adjustments, trade restrictions, and sudden policy events. Consequently, compared with methods relying on historical trends or static fusion strategies, the proposed framework more accurately characterizes short-term direction changes jointly driven by multisource cross-border factors, resulting in more stable and reliable predictions.

### 4.3. Short-Term Volatility Forecasting and Cross-Border Risk Prediction

This experiment was designed to evaluate the comprehensive performance of different models for short-term volatility forecasting and cross-border risk prediction, with particular emphasis on their capability to model continuous state variations and identify high-risk events. Compared with direction prediction, volatility forecasting focuses more strongly on uncertainty and the magnitude of price disturbances, whereas risk prediction requires abnormal states to be identified at an early stage. Therefore, this experiment provides a further assessment of the ability of different methods to perceive complex cross-border shocks.

**Figure 5:**
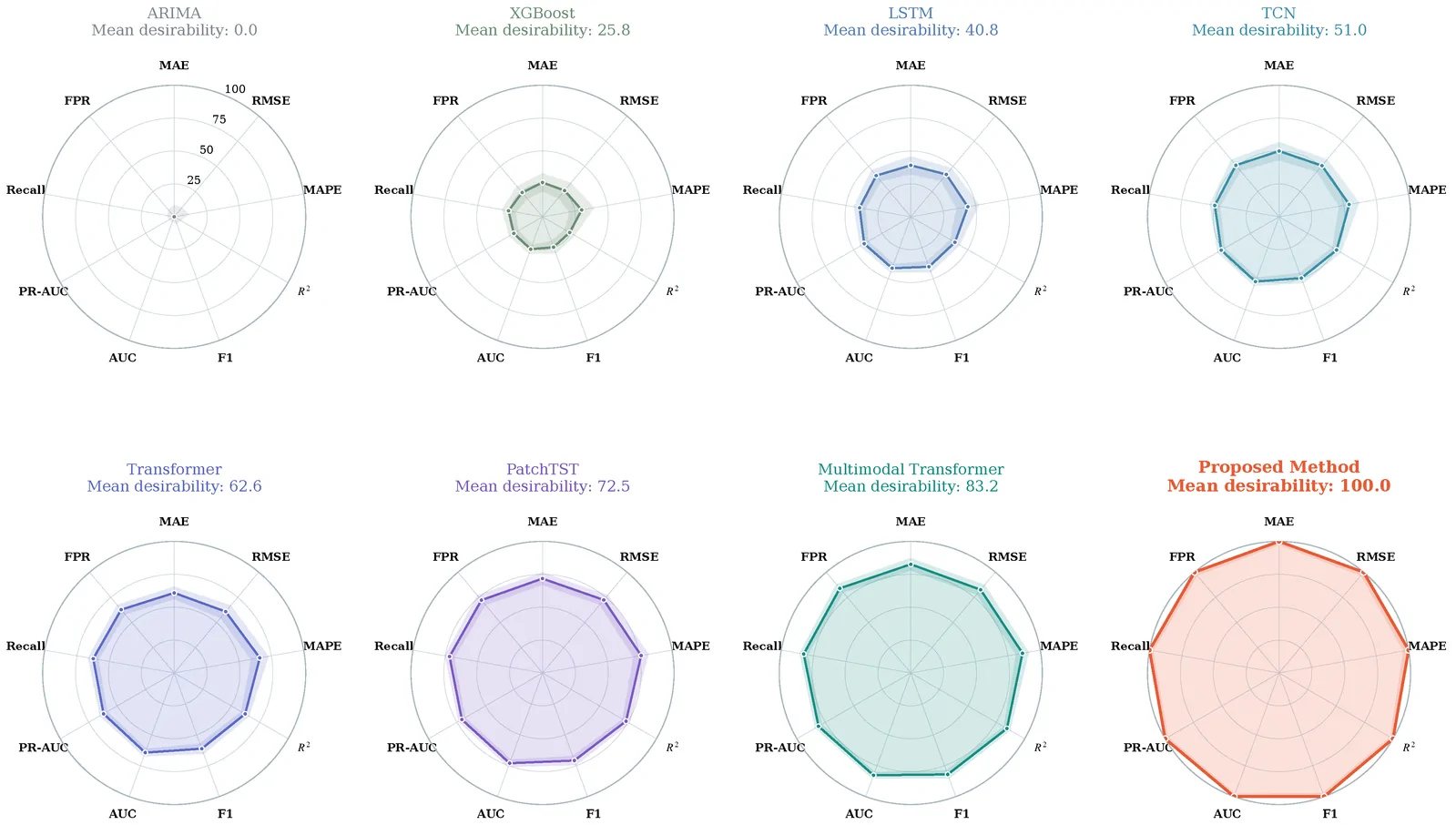
Performance comparison on short-term volatility forecasting and cross-border risk prediction.

As shown in Table 3 and Figure 5, the experimental results show that ARIMA exhibited relatively limited performance in both tasks. For volatility forecasting, its MAE, RMSE, and MAPE were 0.084, 0.126, and 18.7, respectively, while the macro-F1 and AUC values for risk prediction reached only 0.584 and 0.651. This limitation is mainly attributable to the linear stationarity assumptions of ARIMA, where future variations are inferred from autocorrelation structures within historical sequences. Consequently, non-stationary volatility induced by sudden policy changes, logistics anomalies, and cross-border supply-chain disruptions cannot be effectively accommodated. XGBoost improved predictive performance through nonlinear ensembles of decision trees, increasing $R^{2}$ to 0.681 for volatility forecasting and AUC to 0.721 for risk prediction. These results indicate that tree-based feature partitioning can capture complex mappings between variables and risk states. However, because static feature representations are primarily employed, the dynamic evolution of risk caused by temporally propagating cross-border events remains difficult to model. LSTM further improved performance, reducing MAE to 0.066 and increasing risk AUC to 0.762. Its gated recurrent structure enables long-term historical information to be retained, thereby providing improved modeling capability for continuous volatility. TCN achieved further improvements, with MAE decreasing to 0.061 and AUC increasing to 0.791. Its causal and dilated convolutional structure enables local fluctuation patterns and longer-term temporal trends to be extracted simultaneously, resulting in stronger performance for short-term risk state identification.

Attention-based architectures exhibited further advantages in complex environments. Transformer increased the AUC for risk prediction to 0.823, primarily because self-attention establishes global relationships across different temporal positions and thereby captures long-range dependencies. PatchTST further achieved an MAE of 0.051 and an $R^{2}$ of 0.824. Its patch-based representation reduces the difficulty of long-sequence modeling and facilitates simultaneous learning of local volatility structures and long-term trends. After integrating trade, logistics, and textual event information, Multimodal Transformer achieved an MAE of 0.046 and an AUC of 0.872, indicating that external cross-border state information provides valuable complementary evidence for risk assessment. The proposed method achieved the best overall performance, with MAE, RMSE, and MAPE values of 0.038, 0.057, and 7.8 for volatility forecasting, together with an $R^{2}$ of 0.901. For risk prediction, macro-F1, AUC, PR-AUC, and recall reached 0.824, 0.918, 0.836, and 0.819, respectively, while FPR was reduced to 0.081. Compared with Multimodal Transformer, the proposed method improved volatility MAE and risk AUC by approximately 17.4% and 5.3%, respectively. These advantages are primarily derived from the multi-level dynamic modeling mechanism developed for cross-border risk formation. Conventional multimodal models generally assume that all input sources contribute with relatively stable reliability, whereas actual cross-border environments may involve delayed trade statistics, missing logistics trajectories, and uncertain news events. Therefore, reliability and consistency modeling are employed to dynamically regulate information-source contributions and reduce interference from abnormal signals. Meanwhile, the asynchronous temporal interaction mechanism exploits differences in information propagation speed to identify lagged relationships among logistics variations, policy shocks, and responses, while the dynamic event perception module strengthens responsiveness to extreme-risk states. From a modeling perspective, attention mechanisms improve long-range dependency representation, whereas reliability constraints and event gating further restrict excessive responses induced by noisy or abnormal inputs. As a result, more stable discrimination between normal fluctuations and extreme-risk conditions is achieved, leading to improved volatility forecasting accuracy and more reliable cross-border risk prediction.

### 4.4. Ablation Study of Different Components

The ablation experiment was designed to quantify the independent contribution of each component to short-term cross-border direction prediction and to verify the necessity of reliability modeling, consistency modeling, asynchronous temporal interaction, event perception, and context-aware fusion under complex cross-border conditions. Different degraded variants were constructed by removing individual components from the complete framework and comparing their performance with that of the full model.

**Figure 6:**
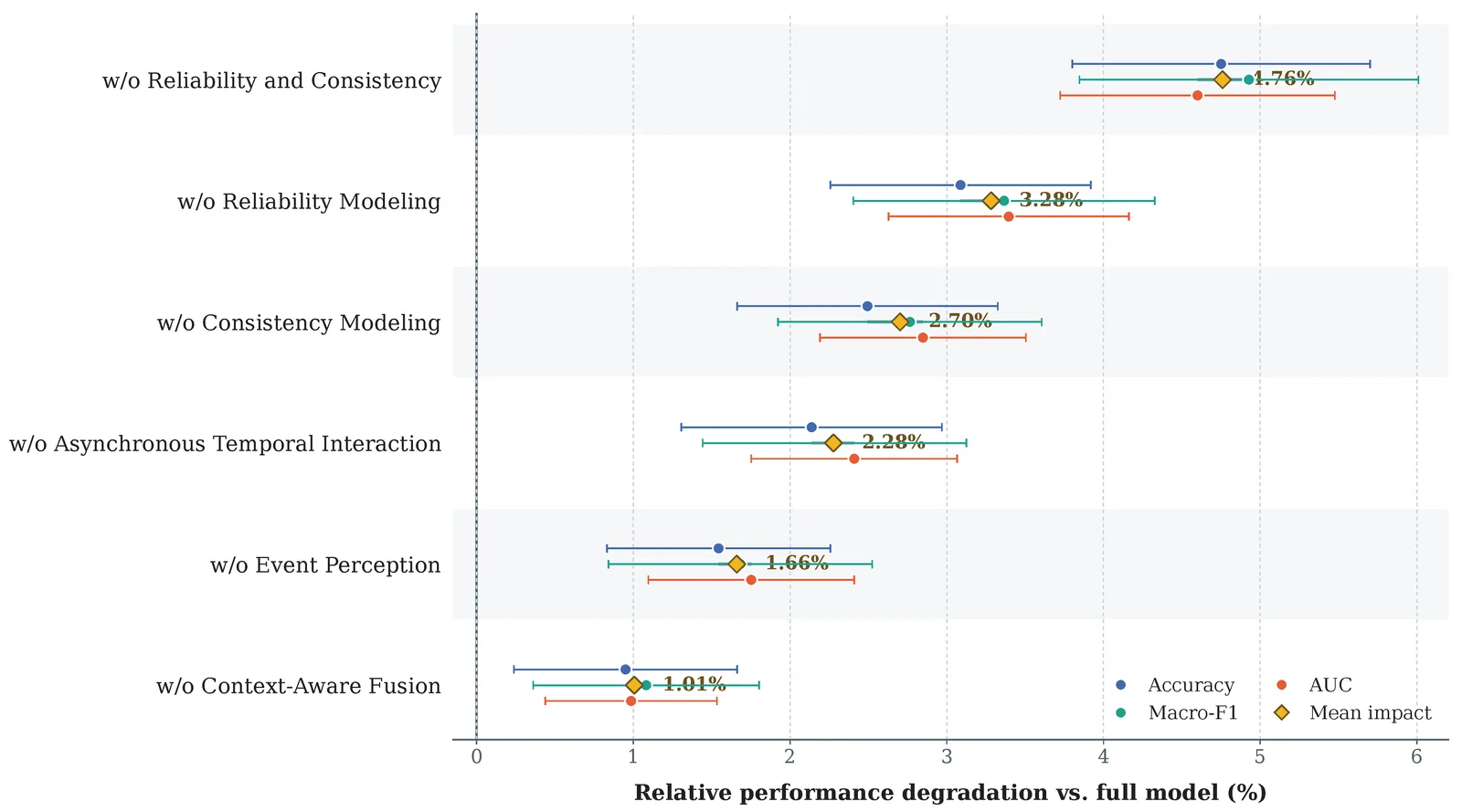
Impact analysis of different components through ablation study.

As shown in Table 4 and Figure 6, the full model achieved the best performance, with accuracy, macro-F1, and AUC values of 0.842, 0.832, and 0.913, respectively, demonstrating clear complementarity among the different components. After reliability modeling was removed, performance decreased to an accuracy of 0.816, a macro-F1 of 0.804, and an AUC of 0.882, indicating that variations in data quality among heterogeneous information sources significantly affect cross-border prediction. Because trade statistics may be delayed, logistics trajectories may be incomplete, and news information may contain noise, direct dependence on raw modality inputs can increase sensitivity to low-quality signals. Reliability estimation therefore contributes to suppressing the negative influence of abnormal information. When consistency modeling was removed, the resulting performance remained slightly higher than that obtained after removing reliability modeling, but accuracy and AUC still decreased to 0.821 and 0.887, respectively. This result demonstrates that cross-modal relational constraints are important for identifying genuine economic transmission relationships. When news, trade, logistics, and signals are mutually aligned, multisource information can provide reinforcing evidence; conversely, consistency assessment helps avoid misleading associations when abrupt changes are observed in only a single modality. When both reliability and consistency mechanisms were removed, the most substantial degradation was observed, with Accuracy decreasing to 0.802 and AUC to 0.871. This finding confirms the synergistic relationship between the two mechanisms and indicates that data quality and cross-modal relationships cannot be treated independently when addressing the complexity of cross-border information.

Removing the asynchronous temporal interaction module reduced accuracy to 0.824 and AUC to 0.891, demonstrating that heterogeneous cross-border information does not influence target simultaneously and that significant propagation delays exist among different signals. Conventional synchronous fusion may distort the actual temporal relationships among trade variation, logistics adjustment, policy release, and response, whereas asynchronous temporal modeling captures differences in propagation speed through explicit temporal dependencies. Removal of the event perception module reduced accuracy and AUC to 0.829 and 0.897, respectively, indicating that sudden events such as tariff adjustments, trade restrictions, and port disruptions play an important role in short-term direction changes. Conventional time-series models mainly learn historical trends and may continue extrapolating normal dynamics under external shocks, whereas event perception allows abnormal disturbances to be identified and corresponding states to be corrected. Removing context-aware fusion produced a relatively smaller performance decline, with accuracy and AUC decreasing to 0.834 and 0.904, respectively, indicating that the relative importance of different modalities varies across regimes and that dynamic information weighting contributes to improved stability. Structurally, the reliability and consistency mechanisms reduce the influence of noisy modalities through dynamic weighting constraints, asynchronous temporal interaction strengthens long-range dependency representation through attention-based modeling, event perception limits and calibrates event-driven disturbances through gating mechanisms, and context-aware fusion further optimizes information combinations under different states. Consequently, the complete framework jointly addresses data credibility, temporal asynchrony, and external shocks in cross-border prediction, resulting in more stable and accurate performance.

### 4.5. Ablation Study on Data Augmentation Strategies

To systematically isolate and quantify the individual contributions of the training-time perturbation mechanisms described in Section 3.3, we conducted a comprehensive leave-one-out ablation study across all three forecast horizons (h∈{15,30,60} minutes). Throughout all variants, the network backbone architecture, random seeds, temporal chronological cross-validation folds, early stopping criteria, and out-of-sample test splits were kept identical to ensure strict comparability. Specifically, the full augmentation pipeline was benchmarked against six single-component removal variants: (1) without modality masking, (2) without temporal masking, (3) without Gaussian feature noise, (4) without causal time shifting, (5) without cross-modal shuffling, and (6) without event-window oversampling. In addition, a completely unaugmented baseline (“w/o Any Augmentation”) was evaluated under the identical training schedule.

As summarized in Table 5, removing any single augmentation component leads to consistent degradation across direction prediction, volatility forecasting, and risk classification. Among individual perturbations, disabling cross-modal shuffling incurs the most noticeable drop in classification discrimination (e.g., direction macro-F1 dropping from 0.832 to 0.814 at h=15), confirming its essential role in providing contrastive supervision for the consistency perception module. Eliminating causal time shifting degrades the model’s tolerance to cross-modal update delays, causing the 60 min volatility MAE to worsen from 0.046 to 0.052. Furthermore, removing event-window oversampling substantially harms risk prediction recall during abrupt turbulence periods, as evidenced by the drop in risk AUC across all horizons. Finally, stripping all augmentation operations simultaneously (“w/o Any Augmentation”) results in the most severe performance collapse across all tasks (direction accuracy falls to 0.796 at h=15 min, and overall risk AUC drops to 0.865). These empirical results substantiate that our causally aligned multisource augmentation strategies synergistically prevent overfitting to high-frequency dominant signals and bolster model resilience under real-world data missingness and asynchronous shocks.

### 4.6. Discussion

The proposed framework for short-term cross-border prediction is not limited to improving predictive accuracy; more importantly, it also attempts to establish explicit relationships between variations and actual cross-border economic activities. In practical international trade scenarios, price movements are rarely driven by individual variables and are instead jointly influenced by commodity flows, logistics conditions, policy environments, and investor expectations. For example, in international energy, agricultural commodity, and industrial raw material, port congestion or shipping route disruptions in major exporting countries may first be reflected in reduced vessel arrivals and increased transportation delays before noticeable price increases or volatility amplification occur. Conventional price-series models generally observe only the subsequent outcome and provide limited insight into the underlying supply-side changes. By integrating AIS vessel trajectories, port operational states, and trade-flow information, potential supply-chain pressure can be perceived before corresponding prices fully respond, thereby providing earlier decision support for inventory adjustment, procurement planning, and risk management. Different types of information also exhibit substantially different propagation speeds in real cross-border trade operations. Taking international agricultural trade as an example, an export restriction imposed by a major exporting country may affect sentiment within several hours through news and policy channels, while exchange-rate fluctuations may rapidly change import costs. By contrast, actual variations in trade quantities and logistics operations usually require a longer period before supply-side effects become observable. The asynchronous cross-border temporal interaction mechanism avoids treating these signals as simultaneous observations and instead identifies their actual influence processes according to heterogeneous propagation pathways. Such a mechanism is more consistent with practical transmission processes involving policy changes, logistics adjustments, supply expectation revisions, and subsequent price responses, thereby enhancing not only predictive performance but also economic interpretability. Data reliability is equally important in practical applications for institutions and cross-border enterprises. AIS information may temporarily become unavailable because of equipment failures, trade statistics may be published with substantial delays, and international news sources may contain unverified or uncertain information. If all sources are treated as equally credible, predictions can easily be distorted by abnormal signals. Reliability- and consistency-aware modeling allows the framework to determine whether heterogeneous information sources mutually support each other. For example, when a commodity supply disruption is reported by news sources while vessel traffic simultaneously decreases, port congestion increases, and related prices begin to respond, the corresponding event can be assigned greater importance. In contrast, when an isolated news report is inconsistent with logistics states, its contribution can be appropriately reduced. Such behavior is more consistent with real-world risk analysis practices.

### 4.7. Limitation and Future Work

Although a multimodal cross-border prediction framework integrating exchange-rate, trade, logistics, and policy-event information has been established, several issues remain to be further investigated. First, the current dataset mainly consists of publicly accessible international information, trade statistics, logistics trajectories, and policy-event data. Although these sources can represent major cross-border economic activity variations, substantial differences remain in data accessibility across countries and regions, and data completeness is still limited for some emerging markets and local trade corridors. In addition, cross-border markets are jointly influenced by macroeconomic conditions, industrial cycles, and changes in international relations. The current framework mainly focuses on short-term dynamic propagation processes, while longer-term structural changes remain insufficiently explored. Future research can further incorporate more real-time and fine-grained cross-border sensing information, including port Internet of Things devices, operational data from supply-chain enterprises, and real-time transportation states, thereby improving sensitivity to localized trade anomalies and supply-chain disruptions. Cross-market causal relationship modeling can also be further investigated so that variations can not only be predicted but the influence pathways among different countries, commodities, and industrial chains can also be more deeply interpreted. In addition, as privacy requirements for global trade data continue to increase, privacy-preserving computation and federated learning can be integrated to support cross-institutional and cross-regional collaborative modeling without directly sharing raw commercial data, thereby enabling the development of a more open, secure, and reliable global cross-border intelligence framework.

## 5. Conclusions

Short-term cross-border prediction plays a crucial role in global supply-chain coordination, international trade risk management, and decision-making. With the increasingly strong interactions among global trade activities, logistics networks, exchange-rate fluctuations, and policy events, conventional prediction approaches that rely solely on historical price sequences are insufficient to fully capture real-time state transitions. To address the challenges of heterogeneous multisource data, varying information reliability, asynchronous cross-modal propagation, and difficult modeling of sudden event impacts, a reliability- and consistency-aware multimodal cross-border prediction framework is proposed. Conditions, exchange rates, trade flows, logistics states, and news–policy events are uniformly regarded as cross-border sensing signals, enabling end-to-end intelligent analysis from data perception and information filtering to dynamic interaction and prediction. The main innovations are achieved through the construction of three key modules. First, a reliability- and consistency-aware cross-border representation module is designed, where the influence of low-quality or conflicting information is reduced by dynamically evaluating missing patterns, update delays, noise levels, and cross-modal matching relationships among different information sources. Second, an asynchronous cross-border temporal interaction module is developed, where continuous-time representation and multi-scale lag modeling are introduced to capture the actual propagation pathways among trade, logistics, exchange rates, and states, allowing instantaneous impacts and delayed effects of different signals to be effectively distinguished. Furthermore, a dynamic trade and policy event perception module is constructed to jointly analyze tariff adjustments, trade restrictions, port disruptions, and international news events with current conditions, thereby achieving adaptive responses to non-stationary shocks. Experimental results demonstrate that the proposed framework achieves superior performance in short-term cross-border direction prediction, obtaining an accuracy of 84.2%, precision of 83.6%, recall of 82.9%, macro-F1 of 83.2%, and AUC of 91.3%, which significantly outperform conventional statistical models, machine learning approaches, and existing multimodal deep learning methods. In volatility forecasting and risk identification tasks, the proposed model further achieves an MAE of 0.038, an RMSE of 0.057, an $R^{2}$ of 0.901, and an AUC of 0.918, while reducing the risk prediction FPR to 0.081. Ablation experiments further verify the essential contributions of reliability modeling, consistency constraints, asynchronous temporal interaction, and event perception mechanisms to improving model stability and generalization capability. The results demonstrate that artificial intelligence-driven multisource sensing methods can effectively establish connections among trade activities, logistics operations, and dynamics, providing a new technological pathway for intelligent trade monitoring, supply-chain-risk early warning, and cross-border decision support.

## Figures and Tables

**Table 1:** Statistics of the collected cross-border multimodal sensing dataset.

Data Source	Sensor/Data Platform	Data Quantity	Main Features
Market Data	Bloomberg/Exchange API	2,340,000 records	OHLC, volume, return, volatility, order-book
Foreign Exchange Data	International FX Database	1,280,000 records	Exchange rate, currency return, volatility, spread
Cross-Border Trade Data	Customs and Trade Database	186,500 records	Import/export value, quantity, commodity, country
AIS Vessel Data	Automatic Identification System (AIS)	6,420,000 records	Position, speed, trajectory, arrival, departure
Port Operation Data	Port Monitoring Platform	428,600 records	Throughput, congestion, delay, container flow
Logistics Tracking Data	GPS Logistics Tracking System	1,960,000 records	Route, transportation time, delay, activity index
News and Policy Events	International News Database	96,800 records	Event type, country, commodity, intensity, sentiment
Policy Announcement Data	Government and Trade Agencies	18,600 records	Tariff, restriction, sanction, regulation change

**Table 2:** Performance comparison of different methods for short-term cross-border direction prediction. Results are reported as mean ± 95% confidence interval.

Method	Accuracy ↑	Precision ↑	Recall ↑	Macro-F1 ↑	AUC ↑
ARIMA	0.621 ± 0.012	0.608 ± 0.015	0.596 ± 0.013	0.601 ± 0.014	0.672 ± 0.011
XGBoost	0.694 ± 0.010	0.681 ± 0.012	0.673 ± 0.011	0.676 ± 0.010	0.748 ± 0.009
LSTM	0.716 ± 0.011	0.704 ± 0.013	0.698 ± 0.012	0.700 ± 0.011	0.773 ± 0.010
TCN	0.731 ± 0.009	0.719 ± 0.010	0.712 ± 0.011	0.715 ± 0.009	0.791 ± 0.008
Transformer	0.748 ± 0.010	0.736 ± 0.011	0.729 ± 0.010	0.732 ± 0.010	0.814 ± 0.009
PatchTST	0.761 ± 0.009	0.752 ± 0.010	0.741 ± 0.009	0.746 ± 0.010	0.829 ± 0.008
Early Fusion	0.772 ± 0.008	0.764 ± 0.009	0.755 ± 0.010	0.759 ± 0.009	0.842 ± 0.007
Multimodal Transformer	0.791 ± 0.008	0.783 ± 0.009	0.776 ± 0.008	0.779 ± 0.008	0.861 ± 0.007
Cross-Modal Attention Network	0.803 ± 0.007	0.796 ± 0.008	0.788 ± 0.008	0.792 ± 0.007	0.874 ± 0.006
**Proposed Method**	**0.842 ± 0.006**	**0.836 ± 0.007**	**0.829 ± 0.006**	**0.832 ± 0.006**	**0.913 ± 0.005**

Bold indicates the best results.

**Table 3:** Performance comparison on short-term volatility forecasting and cross-border risk prediction. Results are reported as mean ± 95% confidence interval.

Method	MAE ↓	RMSE ↓	MAPE ↓	$R^{2}$↑	Macro-F1 ↑	AUC ↑	PR-AUC ↑	Recall ↑	FPR ↓
ARIMA	0.084 ± 0.004	0.126 ± 0.006	18.7 ± 1.2	0.612 ± 0.015	0.584 ± 0.012	0.651 ± 0.010	0.562 ± 0.011	0.571 ± 0.013	0.218 ± 0.009
XGBoost	0.072 ± 0.003	0.108 ± 0.005	15.4 ± 1.0	0.681 ± 0.012	0.643 ± 0.011	0.721 ± 0.009	0.631 ± 0.010	0.636 ± 0.012	0.185 ± 0.008
LSTM	0.066 ± 0.003	0.097 ± 0.004	13.9 ± 0.9	0.724 ± 0.011	0.681 ± 0.010	0.762 ± 0.008	0.674 ± 0.009	0.669 ± 0.010	0.162 ± 0.007
TCN	0.061 ± 0.003	0.091 ± 0.004	12.8 ± 0.8	0.758 ± 0.010	0.703 ± 0.009	0.791 ± 0.008	0.701 ± 0.008	0.694 ± 0.009	0.148 ± 0.007
Transformer	0.056 ± 0.002	0.084 ± 0.004	11.5 ± 0.7	0.792 ± 0.009	0.731 ± 0.009	0.823 ± 0.007	0.732 ± 0.008	0.726 ± 0.009	0.132 ± 0.006
PatchTST	0.051 ± 0.002	0.076 ± 0.003	10.4 ± 0.6	0.824 ± 0.008	0.754 ± 0.008	0.846 ± 0.007	0.756 ± 0.007	0.749 ± 0.008	0.119 ± 0.006
Multimodal Transformer	0.046 ± 0.002	0.069 ± 0.003	9.3 ± 0.5	0.856 ± 0.007	0.781 ± 0.007	0.872 ± 0.006	0.784 ± 0.007	0.776 ± 0.007	0.103 ± 0.005
**Proposed Method**	**0.038 ± 0.001**	**0.057 ± 0.002**	**7.8 ± 0.4**	**0.901 ± 0.006**	**0.824 ± 0.006**	**0.918 ± 0.005**	**0.836 ± 0.006**	**0.819 ± 0.006**	**0.081 ± 0.004**

Bold indicates the best results.

**Table 4:** Ablation study of different components in the proposed framework. Results are reported as mean ± 95% confidence interval.

Variant	Accuracy ↑	Macro-F1 ↑	AUC ↑
w/o Reliability Modeling	0.816 ± 0.007	0.804 ± 0.008	0.882 ± 0.007
w/o Consistency Modeling	0.821 ± 0.007	0.809 ± 0.007	0.887 ± 0.006
w/o Reliability and Consistency	0.802 ± 0.008	0.791 ± 0.009	0.871 ± 0.008
w/o Asynchronous Temporal Interaction	0.824 ± 0.007	0.813 ± 0.007	0.891 ± 0.006
w/o Event Perception	0.829 ± 0.006	0.818 ± 0.007	0.897 ± 0.006
w/o Context-Aware Fusion	0.834 ± 0.006	0.823 ± 0.006	0.904 ± 0.005
**Full Model**	**0.842 ± 0.006**	**0.832 ± 0.006**	**0.913 ± 0.005**

Bold indicates the best results.

**Table 5:** Ablation results of data augmentation components across 15, 30, and 60 min prediction horizons. Results are reported as mean ± 95% confidence interval. Best results are marked in bold.

Augmentation Variant	15 min Horizon			30 min Horizon			60 min Horizon		
	Dir. Acc ↑	Vol. MAE ↓	Risk AUC ↑	Dir. Acc ↑	Vol. MAE ↓	Risk AUC ↑	Dir. Acc ↑	Vol. MAE ↓	Risk AUC ↑
w/o Modality Masking	0.828±0.007	0.041±0.002	0.903±0.006	0.817±0.007	0.046±0.002	0.892±0.006	0.803±0.008	0.050±0.003	0.879±0.007
w/o Temporal Masking	0.831±0.006	0.040±0.002	0.907±0.005	0.820±0.007	0.044±0.002	0.896±0.006	0.806±0.007	0.049±0.002	0.883±0.006
w/o Gaussian Noise	0.835±0.006	0.040±0.002	0.910±0.005	0.824±0.006	0.045±0.002	0.898±0.005	0.810±0.007	0.048±0.002	0.887±0.006
w/o Causal Time Shifting	0.823±0.007	0.042±0.002	0.901±0.006	0.812±0.008	0.047±0.003	0.889±0.006	0.798±0.008	0.052±0.003	0.874±0.007
w/o Cross-Modal Shuffling	0.821±0.007	0.043±0.002	0.898±0.006	0.810±0.008	0.048±0.003	0.885±0.006	0.795±0.008	0.053±0.003	0.871±0.007
w/o Event Oversampling	0.833±0.006	0.041±0.002	0.895±0.006	0.821±0.007	0.046±0.002	0.882±0.006	0.808±0.007	0.049±0.002	0.869±0.007
w/o Any Augmentation	0.796±0.009	0.048±0.003	0.865±0.008	0.785±0.009	0.054±0.003	0.852±0.008	0.771±0.010	0.059±0.003	0.839±0.008
**Full Pipeline (Ours)**	**0.842 ± 0.006**	**0.038 ± 0.001**	**0.918 ± 0.005**	**0.831 ± 0.006**	**0.042 ± 0.002**	**0.907 ± 0.005**	**0.819 ± 0.006**	**0.046 ± 0.002**	**0.896 ± 0.005**

## Data Availability

The data presented in this study are available on request from the corresponding author.
